# Extracellular vesicle-mediated spinal cord-brain crosstalk induces hippocampal neurogenesis impairment and cognitive deficits post-spinal cord injury

**DOI:** 10.7150/thno.110560

**Published:** 2025-06-23

**Authors:** Tian Qin, Yiming Qin, Yuxin Jin, Xiangyu Liang, Yi Sun, Baozhi Liu, Hongbin Lu, Chengjun Li, Jianzhong Hu, Liyuan Jiang

**Affiliations:** 1 Department of Spine Surgery and Orthopaedics, Xiangya Hospital, Central South University, Xiangya Road 87, Changsha 410008, China.; 2 Department of Sports Medicine, Research Centre of Sports Medicine, Xiangya Hospital, Central South University, Xiangya Road 87, Changsha 410008, China.; 3 Key Laboratory of Organ Injury, Aging and Regenerative Medicine of Hunan Province, Xiangya Road 87, Changsha 410008, China.; 4 National Clinical Research Center for Geriatric Disorders, Xiangya Hospital, Central South University, Xiangya Road 87, Changsha 410008, China.; 5 Hunan Engineering Research Center of Sports and Health, Changsha 410008, China.

**Keywords:** spinal cord injury, cognitive function, extracellular vesicles, miR-152-3p, microglia, WNT10b, hippocampal neurogenesis

## Abstract

**Rationale:** Spinal cord injury (SCI) is well-documented for its devastating impact on motor and sensory functions. However, its potential effects on cognitive function remain underexplored. This study aims to investigate the mechanisms of SCI-induced cognitive dysfunction, focusing on spinal cord-hippocampal communication mediated by extracellular vesicles (EVs).

**Methods:** Cognitive function and hippocampal neurogenesis were assessed in mice subjected to either SCI or sham surgery. EVs were isolated from spinal cord tissues of SCI and sham groups and stereotactically injected into the hippocampus to evaluate their effects on cognition and neurogenesis. Cx3cr1-CreERT2 transgenic mice combined with AAV-CD63-EGFP injection were used to confirm the source of EVs. High-throughput sequencing was performed to identify differentially expressed miRNAs in EVs from SCI versus sham groups, with miR-152-3p selected for further analysis. RNA sequencing and dual-luciferase reporter assays were used to confirm whether miR-152-3p regulates cognition and neurogenesis via the WNT10b pathway. Finally, stereotactic injection of a WNT agonist was performed to assess its potential for restoring cognition and neurogenesis post-SCI.

**Results:** This study demonstrates that SCI induces cognitive decline and impairs hippocampal neurogenesis in the dentate gyrus (DG) of mice. microglia-derived EVs were identified as critical mediators of communication between the spinal cord and hippocampus. Specifically, microglia-derived EVs were found to carry miR-152-3p, which inhibits WNT10b signaling, disrupts neurogenesis in the DG, and contributes to post-SCI cognitive deficits. Notably, activation of the WNT pathway in hippocampal neural stem cells (NSCs) after SCI promoted neurogenesis and significantly improved cognitive function in SCI mice.

**Conclusion:** This study uncovers a novel microglia-derived EV-mediated communication axis between the spinal cord and hippocampus following SCI. It identifies the miR-152-3p/WNT10b axis as a key regulator of SCI-induced cognitive dysfunction and impaired neurogenesis. Activation of the WNT pathway was shown to restore neurogenesis and cognitive function, providing valuable insights into therapeutic strategies for SCI-associated cognitive impairments.

## Introduction

Spinal cord injury (SCI) is a severe disease of central nervous system resulting in axonal disruption and a series of secondary injuries, such as inflammation and increased vascular permeability, and ultimately loss of motor and sensory function 1, 2. Besides the functional loss of motor-sensory, reports about long-term cognitive impairments in humans after SCI were gaining increasing recognition as a significant clinical concern within the medical and scientific spheres 3-12. In the 1980s, researchers observed that SCI patients might exhibit cognitive deficits. However, due to the limited diagnostic tools and research conditions at the time, these cognitive impairments were primarily attributed to concurrent traumatic brain injuries (TBI). Subsequent imaging studies confirmed that traumatic SCI often co-occurs with brain injuries 13. Even after excluding patients with TBI, cognitive dysfunction was still observed in some SCI patients 14. Roth and colleagues reported that the incidence of cognitive impairments in SCI patients without a history of TBI remained between 10% and 40% across various tests, with neurocognitive test results showing deficits in memory and executive functions compared to non-injured age- and gender-matched controls 15.

At present, the academic community generally believes that cognitive-emotional disorders caused by SCI are mainly due to SCI-induced significant brain reorganization and atrophy in the sensorimotor cortex and corticospinal tract 16-18. These changes are associated with axonal and myelin integrity loss, reflecting the neuroplastic and neurodegenerative responses to spinal cord trauma 19, 20. And some researches had concentrated on the neuroinflammatory responses triggered by spinal cord injury, highlighting the potential for these injuries to instigate inflammatory infiltrates and gliosis within the cerebral cortex and specific nuclear regions 21. Yun Li's research has shown that in a rodent model of SCI, microglia in the cerebral cortex, thalamus, and hippocampus become activated, transitioning from a resting to an activated state characterized by bushy and hypertrophic morphology. This activation is associated with an M1 pro-inflammatory cytokine profile and is present in both acute and chronic phases post-injury, leading to chronic increases in pro-inflammatory cytokines and cell cycle genes in the hippocampus 22. Additionally, Jure's findings reveal a chronic increase in hypertrophic and bushy microglia in the hippocampal dentate gyrus (DG) following severe SCI and provide initial evidence of astrocyte activation in the DG, indicating a role for astrocytes in the neuroinflammatory response alongside microglial cells 9, 23.

However, these researches still primarily focused on the direct pathological changes in the cortex and hippocampus following SCI, with a lack of studies on the mechanistic circuitry between the brain and spinal cord. Multiple factors, such as accelerated cognitive aging, neuroinflammation, and compromised cerebral perfusion, could contribute to the cognitive effects of spinal cord injury (SCI), with a notable impact on attention and executive functioning due to deafferentation and cortical reorganization 24-26. Further research is needed to understand the precise relationships between cognitive impairments and SCI, considering the potential for additional brain injuries and the complex interplay of psychological and physiological factors.

In this study, it was observed that activated microglia in the lesion area released a large number of extracellular vesicles. These activating microglia produced EVs that carry miR-152-3p, which could target the NSCs in the hippocampus and impaired neurogenesis, impacting the cognitive functions. WNT10b serves as a crucial regulatory point in this pathological mechanism; activating the WNT pathway in hippocampal NSCs post-SCI is beneficial for the improvement of cognitive behavioral performance in mice with SCI. Our findings have established a clear link between the damaged spinal cord and hippocampus.

## Results

### Impaired cognition and hippocampal neurogenesis were detected in SCI mice

**Figure 1:**
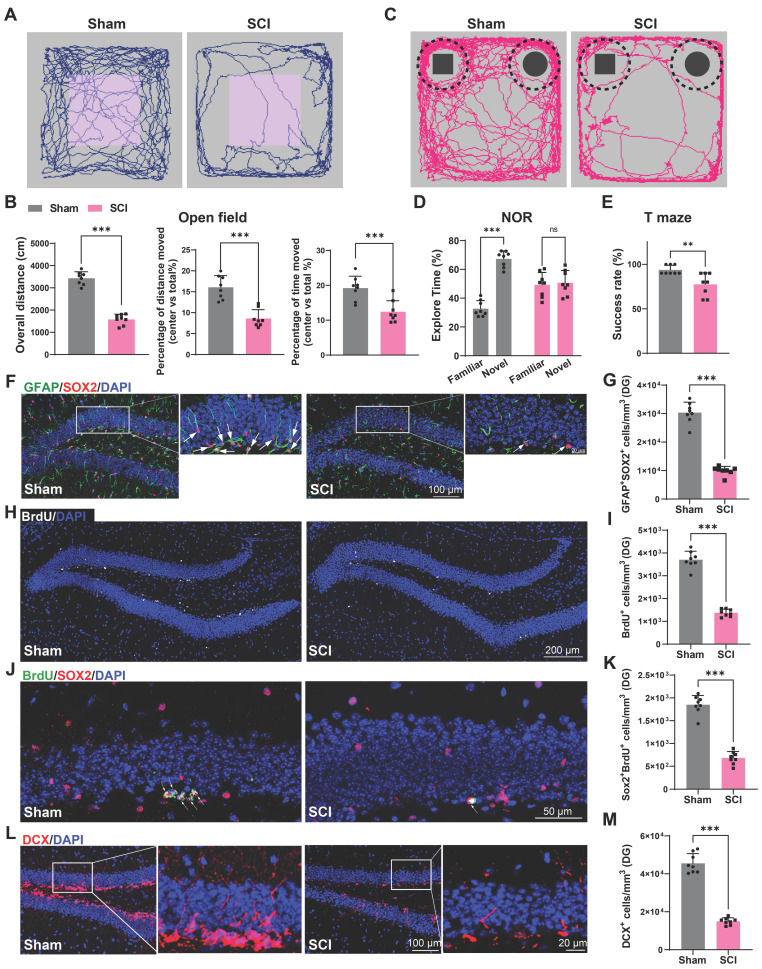
** Impaired cognition and hippocampal neurogenesis were detected in SCI mice.** (A) Trajectory plots illustrating mice movement during the open field test. (B) Quantification mice total move distance (Unpaired t test, *t* = 13.99, *p* < 0.001, *n* = 8), percentage of move distance in center (Unpaired t test, *t* = 6.019, *p* < 0.001, *n* = 8) and percentage of time spent in the central region (Unpaired t test, *t* = 4.147, *p* < 0.001, *n* = 8). (C) Trajectory plots illustrating mice movement during the novel objection reorganization test. (D) Proportion of time spent with the novel or familiar objections. (Unpaired t test, sham vs SCI, *t* = 4.611, *p* < 0.001, *n* = 8) (E) Quantification of success rate of spontaneous alternation of mice in T maze. (Unpaired t test, sham vs SCI, *t* = 3.325, *p* < 0.01, *n* = 8). (F) Representative immunofluorescent stains of the radial glia-like cells (GFAP, green fluoresce, SOX2, Red fluoresces) images of the hippocampus at 8 weeks post-injury in each group. Scale bar, 100μm. (G) Quantification of GFAP^+^SOX2^+^ cells of sham and SCI groups in (F). (Unpaired t test, *t* = 14.53, *p* < 0.001, *n* = 8). (H) Representative immunofluorescent stains of the BrdU^+^ cells (BrdU, white fluoresces) images of the hippocampus at 8 weeks post-injury in each group. Scale bar, 200 μm. (I) Quantification of BrdU^+^ cells of sham and SCI groups in (H). (Unpaired t test, *t* = 16.35, *p* < 0.001, *n* = 8). (J) Representative immunofluorescent stains of the SOX2^+^BrdU^+^ cells (SOX2, Red fluoresces, BrdU, green fluoresces) images of the hippocampus at 8 weeks post-injury in each group. Scale bar, 50 μm. (K) Quantification of SOX2^+^BrdU^+^ cells of sham and SCI groups in (J). (Unpaired t test, *t* = 13.39, *p* < 0.001, *n* = 8). (L) Representative immunofluorescent stains of the neuroblast (DCX, Red fluoresces) images of the hippocampus at 8 weeks post-injury in each group. Scale bar, 100 μm. (M) Quantification of DCX^+^ cells of sham and SCI groups in (L). (Unpaired t test, *t* = 15.88, *p* < 0.001, *n* = 8). Data are presented as mean ± SD, NS, no significant difference, *P < 0.05, **P < 0.01, ***P < 0.001.

We first established a T10 contusion model in mice, and validated its successful establishment through Basso Mouse Scale (BMS) score and histological assessment (Figure S1A-D). Cognitive function was evaluated at 56 days post-injury in both SCI and sham groups using open field, novel object recognition (NOR), and T-maze tests. The open field test revealed significant reductions in overall movement and central area occupancy in SCI mice, which is indicative of depression-like and anxiety behaviors (Figure 1A-B). The NOR test showed a diminished interest in novel objects, suggesting impairments in recognition memory in SCI mice (Figure 1C-D). The T-maze outcomes indicated a decreased likelihood of selecting the correct arm in SCI mice, reflecting declines in learning memory (Figure 1E). Since adult hippocampal neurogenesis, which has a direct impact on cognitive function, has been widely studied in mammalian systems, the impact of SCI on adult hippocampal neurogenesis were assessed 27, 28. 56 days post-SCI, a significant reduction in BrdU^+^ and SOX2^+^GFAP^+^ radial glia-like (RGL) cells was observed in the sub-granular zone (SGZ) of the dentate gyrus (DG) (Figure 1F-I). Furthermore, a marked decrease in SOX2^+^BrdU^+^ cells in the SGZ of SCI mice suggested a curtailed self-renewal capacity of neural stem cells (NSCs) (Figure 1J-K). Consistently, a reduction in DCX^+^ immature neurons was noted, indicative of suppressed hippocampal neurogenesis (Figure 1L-M). In conclusion, SCI could result in a reduced NSCs pool and impaired hippocampal neurogenesis, culminating in cognitive deficits.

### EVs derived from SCI tissue inhibited neurogenesis in the hippocampus, leading to cognitive dysfunction in mice

**Figure 2:**
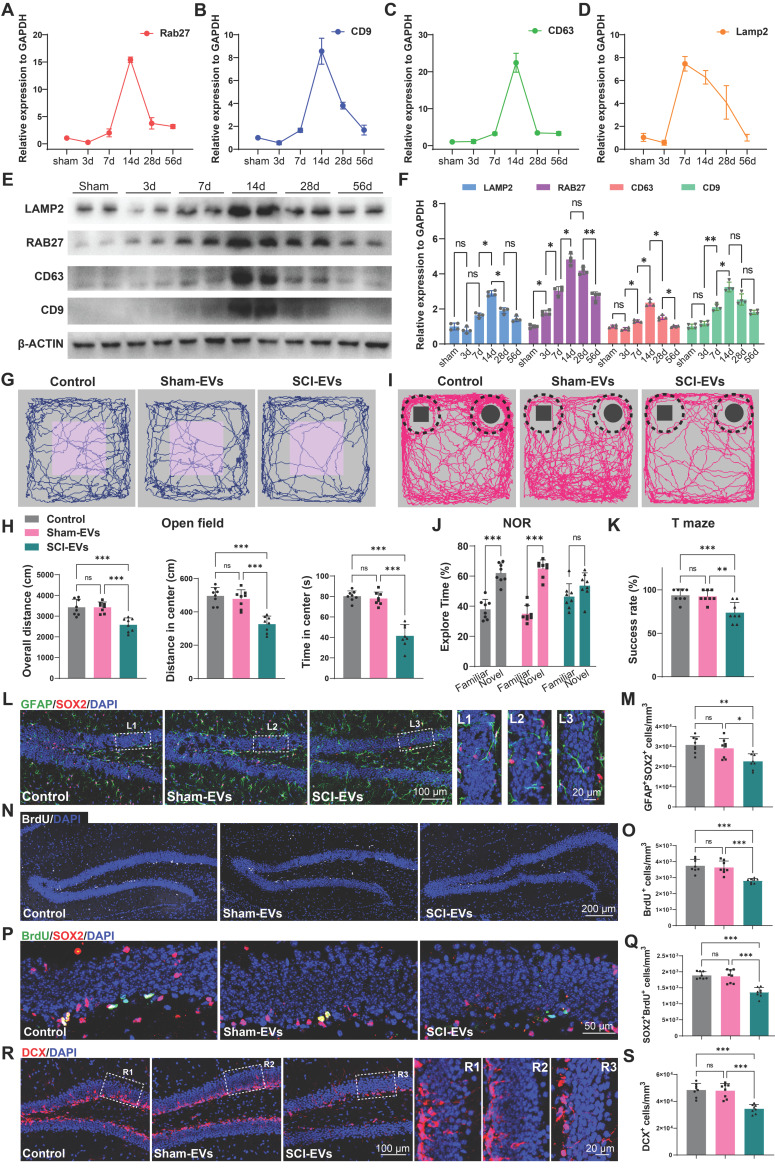
** EVs derived from SCI tissue inhibited neurogenesis in the hippocampus, leading to cognitive dysfunction in mice.** (A-D) Quantitative analysis of the Rab27, CD9, CD63, Lamp2 expression in spinal cord at 3, 7, 14, 28, 56 days post injury using qRT-PCR. N = 5 per group. (E) Western blot analysis of RAB27, CD9, CD63, LAMP2 in spinal cord of sham and SCI groups at 3, 7, 14, 28, 56 days post injury. (F) Quantification of RAB27, CD9, CD63, LAMP2 expression in (E). (two-way ANOVA, *F* (3, 16) = 586.4, *p* < 0.01. Tukey's *post hoc* test. *n* = 4 per group). (G) Trajectory plots illustrating mice movement during the open field test. (H) Quantification mice total move distance (one-way ANOVA, *F (2, 21) = 17.78*, *p* < 0.001. Tukey's *post hoc* test. *n* = 8 per group), move distance in center (one-way ANOVA, *F (2, 21) = 26.43*, *p* < 0.001. Tukey's *post hoc* test. *n* = 8 per group) and time spent in the central region (one-way ANOVA, *F (2, 21) = 35.99*, *p* < 0.001. Tukey's *post hoc* test. *n* = 8 per group). (I) Trajectory plots illustrating mice movement during the novel objection reorganization test. (J) Proportion of time spent with the novel or familiar objections (one-way ANOVA, *F (2, 42) = 11.41*, *p* < 0.001. Tukey's *post hoc* test. *n* = 8 per group). (K) Quantification of success rate of spontaneous alternation of mice (one-way ANOVA, *F (2, 21) = 12.22*, *p* < 0.001. Tukey's *post hoc* test. *n* = 8 per group). (L) Representative immunofluorescent stains of the radial glia-like cells (GFAP, green fluoresce, SOX2, Red fluoresces) images of the hippocampus at 8 weeks post-injury in each group. Scale bar, 100 μm. (L1-L3) Enlarged images of the white box area in (L). Scale bar, 20 μm. (M) Quantification of GFAP^+^SOX2^+^ cells of sham and SCI groups in (L) (one-way ANOVA, *F (2, 21) = 8.353*, *p* < 0.001. Tukey's *post hoc* test. *n* = 8 per group). (N) Representative immunofluorescent stains of the BrdU^+^ cells (BrdU, white fluoresces) images of the hippocampus at 8 weeks post-injury in each group. Scale bar, 200 μm. (O) Quantification of BrdU^+^ cells of sham and SCI groups in (N) (one-way ANOVA, *F* (2, 21) = 18.21, *p* < 0.001. Tukey's post hoc test. *n* = 8 per group). (P) Representative immunofluorescent stains of the SOX2^+^BrdU^+^ cells (SOX2, Red fluoresces, BrdU, green fluoresces) images of the hippocampus at 8 weeks post-injury in each group. Scale bar, 50 μm. (Q) Quantification of SOX2^+^BrdU^+^ cells of sham and SCI groups in (P) (one-way ANOVA, *F* (2, 21) = 26.98, *p* < 0.001. Tukey's post hoc test. *n* = 8 per group). (R) Representative immunofluorescent stains of the neuroblast (DCX, Red fluoresces) images of the hippocampus at 8 weeks post-injury in each group. Scale bar, 100 μm. (R1-R3) Enlarged images of the white box area in (L). Scale bar, 20 μm. (S) Quantification of DCX^+^ cells of sham and SCI groups in (R) (one-way ANOVA, *F* (2, 21) = 23.72, *p* < 0.001. Tukey's post hoc test. *n* = 8 per group). Data are presented as mean ± SD, NS, no significant difference, *P < 0.05, **P < 0.01, ***P < 0.001.

Extracellular vesicles (EVs) are membrane-bound vesicles released by cells into the extracellular environment, playing a crucial role in intercellular communication. These vesicles can carry a variety of biomolecules, such as proteins, lipids, mRNA, and miRNA, enabling them to transfer information and substances between different cells or tissues 29-31. To explore the mechanism by which SCI promotes cognitive deficits, we examined the expression of EVs biogenesis and release-related genes in the injured spinal cord at specific time points post-injury. It was observed that the expression of genes associated with EVs production and release (*Rab27, Cd9, Cd63, Lamp2*) increased following SCI, peaking at 14 days post-injury (Figure 2A-D). Consistently, western blot analysis also corroborated this trend, showing an upregulation of the EVs-associated protein (RAB27, CD9, CD63, LAMP2), with expression levels peaking 14 days post SCI (Figure 2E-F). Subsequentially, we extracted the EVs from the spinal tissue from both SCI and Sham mice at 14 days post injury (dpi). The purified EVs were verified by transmission electron microscopy (TEM), NanoSight analysis, and immunoblotting for protein markers (CD9, CD63, and TSG101), according to the proposal of the International Society of Extracellular Vesicles (Figure S2A-C).

To confirm the role of EVs from the sham or injured spinal cord (Sham-EVs or SCI-EVs) in hippocampal neurogenesis, using stereotactic techniques, EVs were directly injected into the hippocampal tissue of the naive male mice. Meanwhile, an equal volume of PBS was also injected into naive male mice as a control. Cognitive function was analysis 56 days after EVs injection. It was observed that the mice treated with SCI-EVs exhibited depressive-like and anxiety alterations (Figure 2G-H) and a decline in their recognition and learning memory capabilities (Figure 2I-K). And a significant reduction in BrdU^+^ and SOX2^+^GFAP^+^ RGL cells was observed in the DG of the SCI-EVs treated mice (Figure 2L-O). Furthermore, a marked decrease in SOX2^+^BrdU^+^ cells in the SGZ of SCI mice suggested a curtailed self-renewal capacity of NSCs in the SCI-EVs treated mice (Figure 2P-Q). Consistently, a reduction in DCX^+^ immature neurons was noted, indicative of suppressed hippocampal neurogenesis after SCI-EVs administration (Figure 2R-S). These results indicated that EVs released from the region of injured spinal cord could suppress neurogenesis in the mouse hippocampus, culminating in cognitive impairments post-SCI.

### EVs released by injured spinal cord carry miR-152-3p, suppressing neurogenesis in hippocampus of the SCI mice

**Figure 3:**
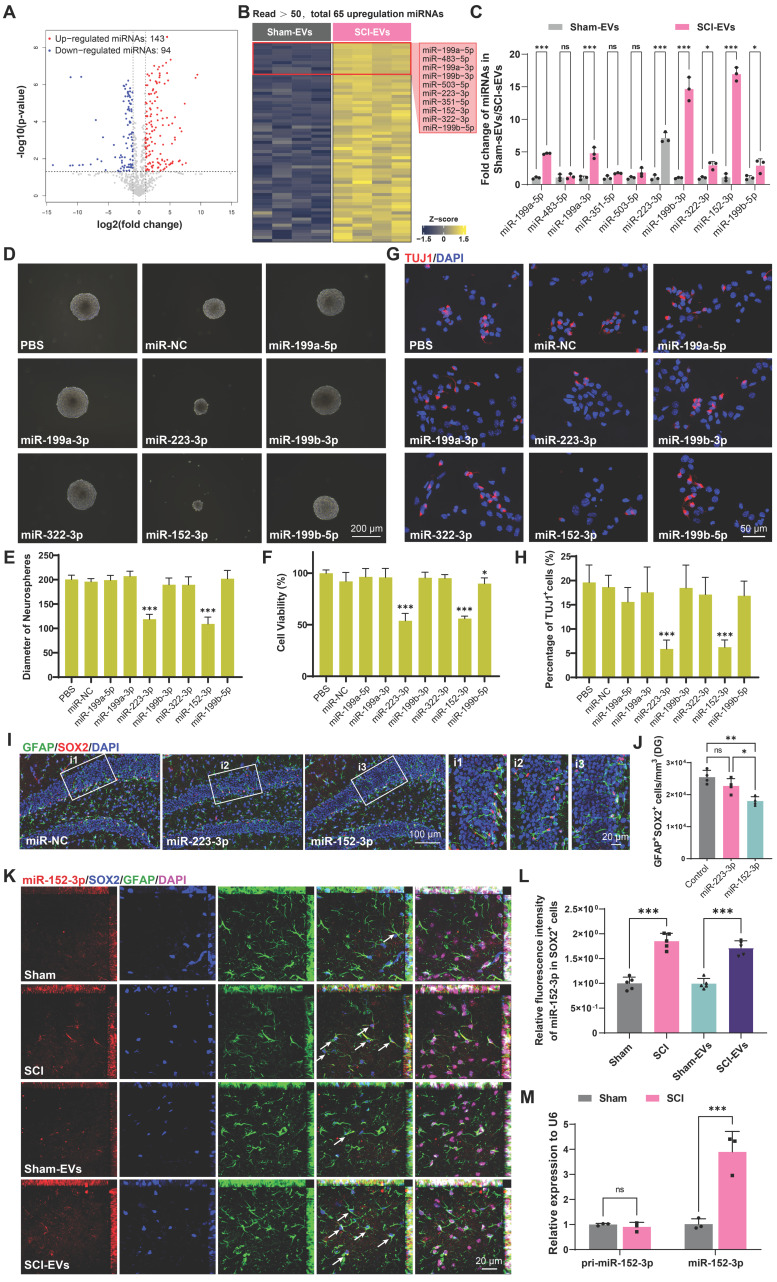
** EVs released by injured spinal cord carrying miR-152-3p could suppress neurogenesis in the mouse hippocampus.** (A) Volcano plot showed the expression pattern of miRNAs from the sham-Evs and SCI-EVs. (B) Heatmap showed the differentially upregulated miRNAs with a certain level of expression abundance (reads > 50). (C) Quantitative analysis of the top10 differentially upregulated miRNAs between sham-Evs and SCI-EVs using qRT-PCR (Unpaired t test, *n* = 3 per group). (D) Neurosphere assay showed the secondary neurospheres size under miRNAs administration. (E) Neural differentiation assay showed the different rate of NSCs under miRNAs administration. (F) Quantitative analysis of average neurosphere diameter in (D) (one-way ANOVA, *F* (8, 153) = 166.4, *p* < 0.001. Tukey's post hoc test. *n* = 18 per group). (G) The survival and cell viability of NSCs co-incubated with miRNAs were detected by the CCK-8 assay (one-way ANOVA, *F* (8, 45) = 48.38, *p* < 0.001. Tukey's post hoc test. *n* = 6 per group). (H) Quantitative analysis of neural different proportion in (E) (one-way ANOVA, *F* (8, 45) = 14.32, *p* < 0.001. Tukey's post hoc test. *n* = 6 per group). (I) Representative immunofluorescent stains of the radial glia-like cells (GFAP, green fluoresce, SOX2, Red fluoresces) images of the hippocampus after miRNAs injection in each group. Scale bar, 100 μm. (i1-i3) Enlarged images of the white box area in (I). Scale bar, 20 μm. (J) Quantification of GFAP^+^SOX2^+^ cells of miR-NC, miR-152-3p and miR-223-3p groups in (I) (one-way ANOVA, *F* (2, 9) = 14.86, *p* < 0.001. Tukey's post hoc test. *n* = 4 per group). (K) Representative fluorescence *in situ* hybridization image of the hippocampus tissue of the sham, SCI, Sham-EVs and SCI-EVs treated mice (GFAP, green fluoresce, SOX2, blue fluoresces, miR-152-3p, red fluoresces). scale bar, 100 μm. (L) Quantification of miR-152-3p immunofluorescent intensity of the GFAP^+^SOX2^+^ cells in (K) (one-way ANOVA, *F* (3, 16) = 57.69, *p* < 0.001. Tukey's post hoc test. *n* = 5 per group). (M) Quantification of pri-miR-152-3p and mature miR-152-3p expression in hippocampus using qRT-PCR (Unpaired t test, *n* = 3 per group). Data are presented as mean ± SD, NS, no significant difference, *P < 0.05, **P < 0.01, ***P < 0.001.

The miRNAs, which can be abundantly encapsulated in EVs, are key regulators of neurogenesis in health and disease 30, 32. To explore how SCI-derived EVs inhibited neurogenesis, the miRNA expression profiles in sham-EVs and SCI-EVs groups were analyzed using miRNA sequencing. To identify the specific miRNAs involved, we compared miRNA profiles of EVs derived from the SCI and the sham mice at 14 days post injury; the results are shown by heatmaps and volcano plot (Figure 3A-B). The top 10 upregulated miRNAs were validated by real-time quantitative polymerase chain reaction (qRT-PCR) (Figure 3C). To determine the effects of these miRNAs on NSCs proliferation and neural differentiation, primary mice NSCs were transfected with miRNA mimics or negative controls. It was found that miR-152-3p and miR-223-3p significantly inhibit NSCs proliferation, which was validated by neurosphere assay and CCK-8 analysis (Figure 3D-F). Consistently, miR-152-3p and miR-223-3p also inhibited the neural differentiation *in vitro* (Figure 3G-H). Using stereotactic techniques, mimics of miR-152-3p and miR-223-3p were injected into the hippocampal of naive male mice, and it was found that only miR-152-3p could significantly reduce the NSCs pool in the hippocampus (Figure 3I-J). Further, we utilized fluorescence *in situ* hybridization (FISH) to analyze the expression and localization of miRNAs associated with NSCs in the hippocampal tissue following SCI. It was observed that the expression of miR-152-3p in hippocampal NSCs was significantly increased after SCI in mice, and an elevated expression of miR-152-3p was also present in the hippocampus of mice following intervention with SCI-EVs (Figure 3K-L). However, using qRT-PCR to assess the expression of mature and precursor forms of miR-152-3p in hippocampal tissue following SCI, it was found that only the mature form of miR-152-3p was upregulated in the hippocampal tissue post-injury, while there was no significant difference in the expression of the miR-152-3p precursor (Figure 3M). These results suggested that the altered expression of miR-152-3p in the hippocampus originates from EVs secreted by the injured spinal cord tissue, leading to a series of abnormal changes in neurogenesis.

### Microglia derived EVs accumulate in the SGZ and could be internalized by hippocampal NSCs

**Figure 4:**
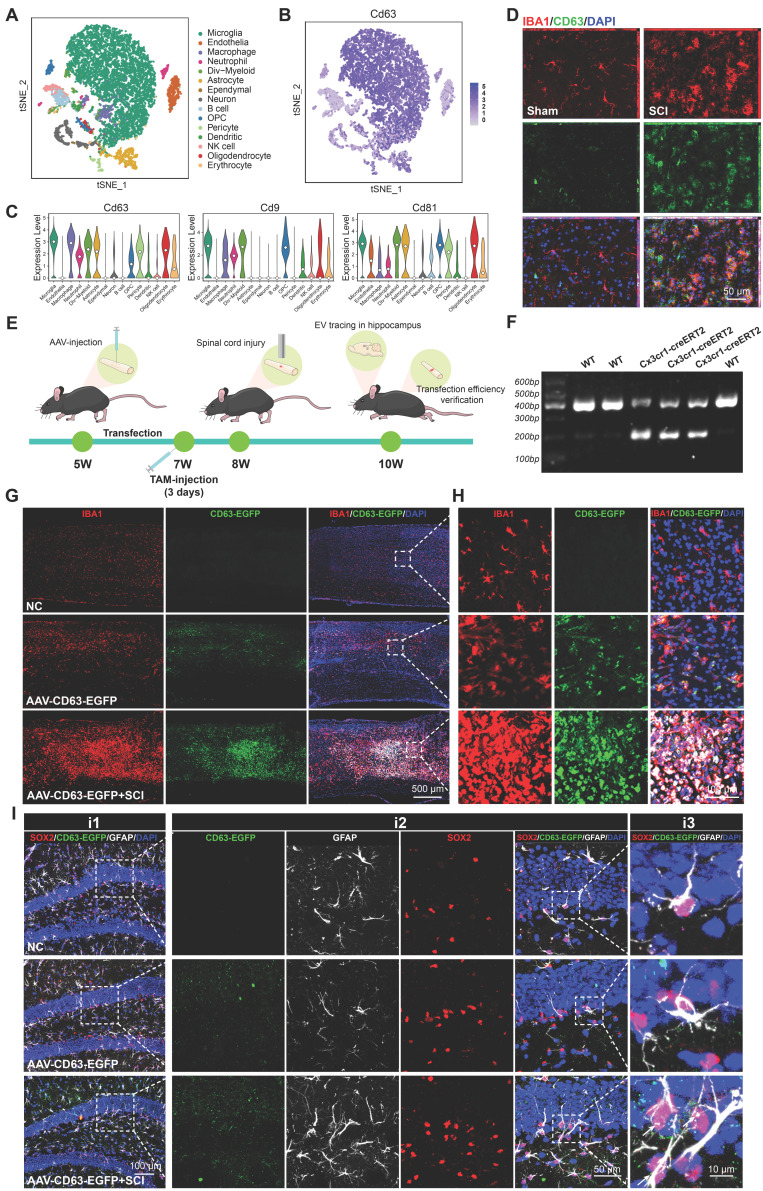
** Microglia derived EVs accumulate in the SGZ and could be internalized by hippocampal NSCs.** (A) t-Distributed Stochastic Neighbor Embedding (t-SNE) map of the cells from mice spinal cord. Colors indicate assigned activation states and cell types. (B) Expression distribution of selected marker genes. (C) Violin plots showing the course of expression for genes in different cell types. (D) Representative immunofluorescent images (CD63, green fluoresce, IBA1, red fluoresces) of the spinal cord from sham or SCI mice. Scale bar, 50 μm. (E) *In vivo* AAV transfection procedure. In Cx3cr1-CreERT2 mice, the T10 spinal cord segment was injected locally with pAAV-MG1.2-DIO-EGFP-P2A-Cd63-3xFLAG-WPRE (AAV-CD63-EGFP) and treated with tamoxifen (TAM) to specifically label microglia-derived EVs with EGFP-positive signals. TAM, Tamoxifen. (F) Genotype identification of the Cx3cr1-CreERT2 mice. (G) Representative immunofluorescent pictures showed AAV-CD63-EGFP exhibited specific labeling of the microglia (IBA1, red fluoresce; CD63-EGFP, green fluoresce) in the injection site. Scale bar, 500 μm. (H) Enlarged images of the white box area in (G) for showing the transfection efficiency of microglia. Scale bar, 500 μm. (I) Representative immunofluorescent pictures showed the CD63-EGFP positive EVs spot (white fluoresce) co-labeling with hippocampal NSCs (GFAP^+^SOX2^+^), white arrows refer to the NSCs colocalization with the CD63-EGFP^+^ EVs spot derived from the transfected microglia in the lesional site of the SCI mice. Scale bar, i1, 100 μm, i2, 50 μm, i3, 10 μm.

To identify the characterization of cells in the injured spinal cord, we downloaded a single cell RNA-seq dataset of T9 spinal cord clamp injury in C57BL/6 male and female mouse 33. Male samples with uninjured and 14 days post injury were collected for further analysis. As shown in Figure 4A, the cells were divided into 15 different clusters and annotated as different cell types. The cluster graph and the violin plot showed that a multitude of EVs biogenesis-related genes (CD9, CD63, CD81) are enriched in microglia (Figure 4B-C). Immunofluorescence assays further revealed that upon microglial activation after SCI, an abundance of CD63-positive signals was detected within the lesioned spinal cord, demonstrating co-localization with IBA1^+^ signals (a specific marker for microglia) (Figure 4D). To investigate the impact of microglia-derived EVs on hippocampal neurogenesis and cognitive function in mice, we bred Cx3cr1-CreERT2 mice for specific labeling and intervention of microglia. In Cx3cr1-CreERT2 mice, the T10 spinal cord segment was injected locally with pAAV-MG1.2-DIO-EGFP-P2A-Cd63-3xFLAG-WPRE (AAV-CD63-EGFP) and treated with tamoxifen (TAM) to specifically label microglia-derived EVs with EGFP-positive signals (Figure 4E-F). Meanwhile, an equal volume of PBS was also injected into spinal cord of naive male mice as the negative control (NC). As shown in Figure 4G-H, the mice treated with AAV-CD63-EGFP exhibited specific microglia labeling, which was also consistent with the activation and aggregation of microglia following SCI. To ensure the labeling efficacy, the EVs derived from the spinal cord were harvested and then labeled with PKH26. The double-positive (PKH26⁺EGFP⁺) spots were considered to be these microglia-derived EVs. Based on the observation using STED super-resolution microscopy, it was detected that along with the activation of microglia after injury, the proportion of these EGFP-labeled EVs also increased, indicating the EVs derived from the injury spinal cord were mainly released by microglia (Figure S3A-B). To verify the communication between spinal cord microglia and hippocampal NSCs, the hippocampus of the transfected mice were harvested. It was observed that the EGFP-labeled EVs accumulated in the SGZ and could be internalized by hippocampal NSCs (Figure 4I).

### Microglia could release miR-152-3p abundant EVs affecting hippocampus neurogenesis and cognitive function of mice after SCI

**Figure 5:**
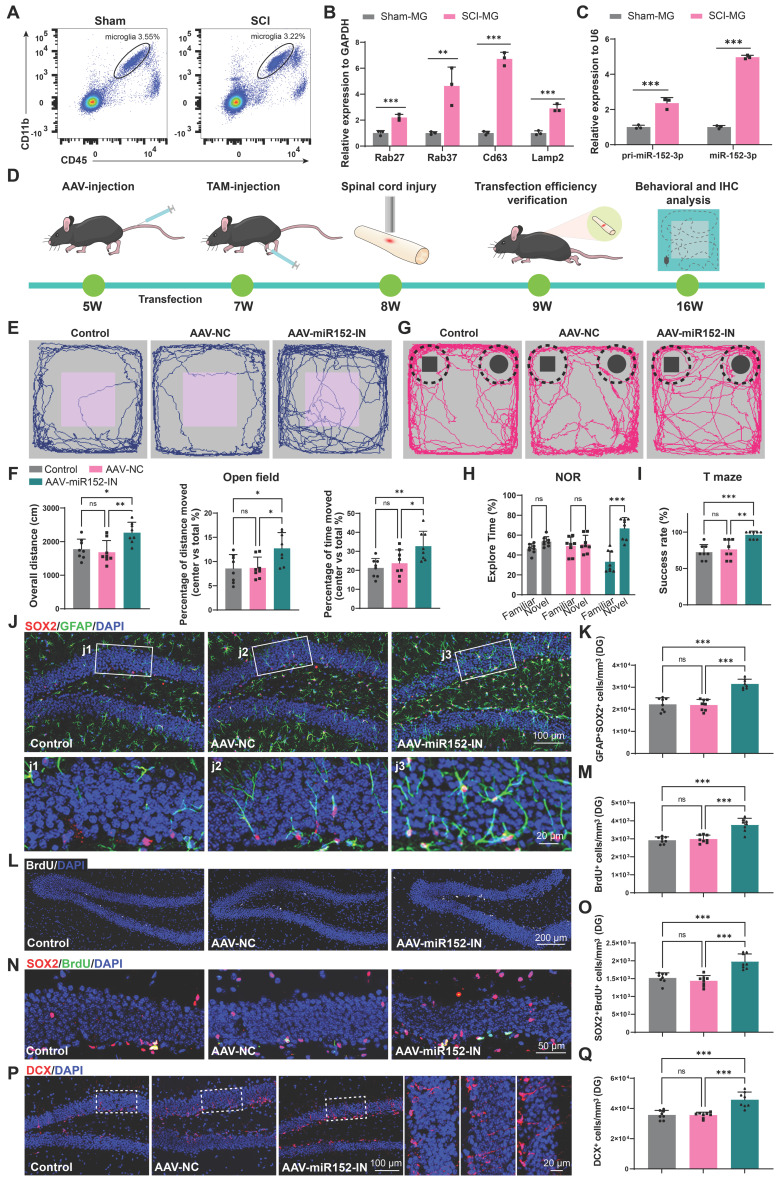
** Microglia can release miR-152-3p abundant EVs affecting hippocampus neurogenesis and cognitive function of mice after SCI.** (A) Flow cytometry sorting of the living CD11b^+^CD45^low^ microglia. (B) Quantification of Rab27, CD63, Lamp2 and Rab27 expression in sorted microglia using qRT-PCR (Unpaired t test, *n* = 3 per group). (C) Quantification of pri-miR-152-3p and mature miR-152-3p expression in sorted microglia using qRT-PCR (Unpaired t test, *n* = 3 per group). (D) *In vivo* AAV transfection procedure. The pAAV-MG1.2-DIO-EGFP-Sponge(mmu-miR-152-3p)-WPRE virus was injected into Cx3cr1-CreERT2 mice via the tail vein. Two weeks after the injection, tamoxifen (TAM) was intraperitoneally injected to induce the expression of Cre enzyme. A spinal cord injury model was established when the mice were 8 weeks old. One week after the injury, the efficiency of virus transfection was verified. Eight weeks after the injury, behavioral tests were conducted, and mouse tissues were obtained for histological section staining. (E) Trajectory plots illustrating mice movement during the open field test. (F) Quantification mice total move distance (one-way ANOVA, *F* (2, 21) = 7.715. *p* = 0.0031 Tukey's post hoc test. *n* = 8 per group), percentage of move distance in center (one-way ANOVA, *F* (2, 21) = 5.653, *p* = 0.0109. Tukey's post hoc test. *n* = 8 per group) and percentage of time spent in the central region (one-way ANOVA, *F* (2, 21) = 6.460, *p* = 0.0065. Tukey's post hoc test. *n* = 8 per group). (G) Trajectory plots illustrating mice movement during the novel objection reorganization test. (H) Proportion of time spent with the novel or familiar objections (one-way ANOVA, *F* (2, 42) = 15.48, *p* < 0.001. Tukey's *post hoc* test. *n* = 8 per group). (I) Quantification of success rate of spontaneous alternation of mice (one-way ANOVA, *F* (2, 21) = 12.89, *p* < 0.001. Tukey's *post hoc* test. *n* = 8 per group). (J) Representative immunofluorescent stains of the radial glia-like cells (GFAP, green fluoresce, SOX2, Red fluoresces) images of the hippocampus at 8 weeks post-injury in each group. Scale bar, 100 μm. (K) Quantification of GFAP^+^SOX2^+^ cells of sham and SCI groups in (J) (one-way ANOVA, *F* (2, 21) = 35.68, *p* < 0.001. Tukey's *post hoc* test. *n* = 8 per group). (L) Representative immunofluorescent stains of the BrdU^+^ cells (BrdU, white fluoresces) images of the hippocampus at 8 weeks post-injury in each group. Scale bar, 200 μm. (M) Quantification of BrdU^+^ cells of sham and SCI groups in (L) (one-way ANOVA, *F* (2, 21) = 25.88, *p* < 0.001. Tukey's *post hoc* test. *n* = 8 per group). (N) Representative immunofluorescent stains of the SOX2^+^BrdU^+^ cells (SOX2, Red fluoresces, BrdU, green fluoresces) images of the hippocampus at 8 weeks post-injury in each group. Scale bar, 50 μm. (O) Quantification of SOX2^+^BrdU^+^ cells of sham and SCI groups in (N) (one-way ANOVA, *F* (2, 21) = 23.60, *p* < 0.001. Tukey's post hoc test. *n* = 8 per group). (P) Representative immunofluorescent stains of the neuroblast (DCX, Red fluoresces) images of the hippocampus at 8 weeks post-injury in each group. Scale bar, 100 μm. (Q) Quantification of DCX^+^ cells of sham and SCI groups in (P) (one-way ANOVA, *F* (2, 21) = 21.15, *p* < 0.001. Tukey's post hoc test. *n* = 8 per group). Data are presented as mean ± SD, NS, no significant difference, *P < 0.05, **P < 0.01, ***P < 0.001.

To verify the role of microglia in the suppression of hippocampal neurogenesis following SCI, we isolated microglia from the injured spinal cord (SCI-MG) or sham mice (Sham-MG) using flow cytometry at 14 days post injury (Figure 5A and Figure S4A). qRT-PCR analysis of these sorted cells also revealed that these microglia express EV-biogenesis-related genes (Figure 5B). And these microglia highly express both the precursor and mature forms of miR-152-3p, suggesting that the high expression of miR-152-3p in EVs derived from the injured spinal cord originates from these activation-induced microglia (Figure 5C). We isolated microglia from the spinal cord and hippocampus of both sham-operated and SCI mice (Figure S5A). qRT-PCR analysis revealed that spinal microglia exhibited significantly elevated miR-152-3p levels following SCI, whereas hippocampal microglia maintained low expression of this microRNA even in SCI mice (Figure S5B). To investigate the impact of microglia-derived miR-152-3p on hippocampal neurogenesis and cognitive function in mice, we bred Cx3cr1-CreERT2 mice for specific labeling and intervention of microglia. Subsequently, the pAAV-MG1.2-DIO-EGFP-Sponge(mmu-miR-152-3p)-WPRE virus (AAV-miR152-IN) were constructed for the specific knockdown of miR-152 in spinal microglia (Figure 5D). The fluorescence co-localization of EGFP (green fluorescence) and IBA1 (red fluorescence) verified the transfection efficiency (Figure S6A). BMS score and cognitive function was analysis after transfection. It was observed that the mice treated with AAV-miR152-IN exhibited no significant improvement in motor function recovery compared to control groups (Figure S7A).

However, these mice treated with AAV-miR152-IN exhibited ameliorated depressive-like and anxiety alterations (Figure 5E-F) and improved recognition and learning memory capabilities (Figure 5G-I). And a significant increase in BrdU^+^ and SOX2^+^GFAP^+^ RGL cells was observed in the DG of the AAV-miR152-IN treated mice (Figure 5J-M). Furthermore, a marked increase in SOX2^+^BrdU^+^ cells in the SGZ of AAV-miR152-IN treated mice suggested an extended self-renewal capacity of NSCs in the AAV-miR152-IN treated mice (Figure 5N-O). Consistently, an increase in DCX^+^ immature neurons was noted, indicative of improved hippocampal neurogenesis after AAV-miR152-IN administration (Figure 5P-Q). These results indicated that microglia-derived miR-152 is a significant factor leading to the decline in neurogenesis and cognitive abilities following SCI, and blocking this pathway can ameliorate these changes.

### Wnt10b in hippocampal NSCs is a key target of EVs-derived miR-152-3p from SCI-activated microglia

**Figure 6:**
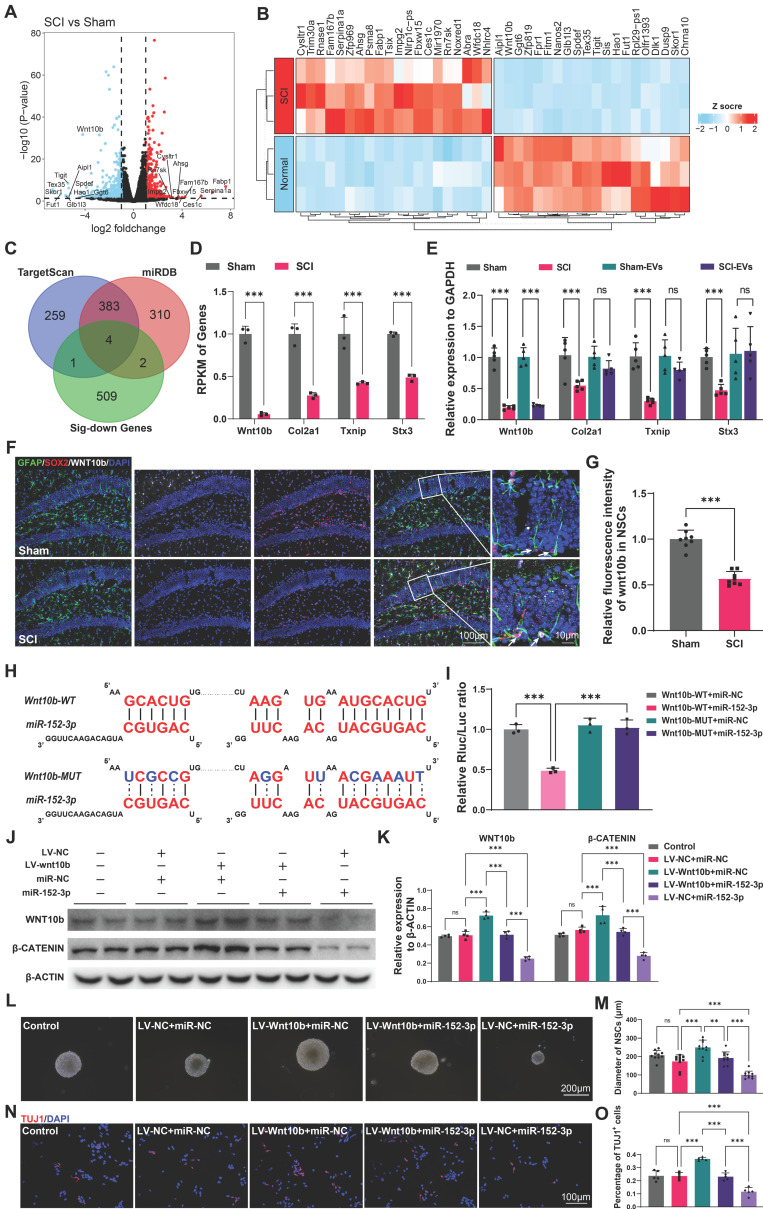
** Wnt10b in hippocampal NSCs is a key target of EVs-derived miR-152-3p from SCI-activated microglia.** (A) Volcano plot showed the expression pattern of mRNAs from the hippocamal NSCs from the sham and SCI groups. (B) Heatmap showed the top 20 differentially exprssion genes between the sham and SCI groups. (C) The Venn diagram demonstrated the potential target genes of miR-152-3p. (D) Reads Per Kilobase per Million mapped reads (RPKM) value of differentially expressed transporter genes in hippocamal NSCs from the sham and SCI groups (Unpaired t test, *n* = 3 per group). (E) Quantitative analysis of the intersection gene expression in hippocampal NSCs between Sham, SCI, Sham-EVs and SCI-EVs groups by qRT-PCR analysis (two-way ANOVA, *F* (9, 48) = 3.407, *p* = 0.0026. Šídák multiple comparison test. *n* = 5 per group). (F) Representative immunofluorescent stains of the radial glia-like cells (GFAP, green fluoresce, SOX2, Red fluoresces, WNT10b, White fluoresces) images of the hippocampus at 8 weeks post-injury in each group. Scale bar, 100 μm. (G) Quantification of WNT10b expression in GFAP^+^SOX2^+^ cells of sham and SCI groups in (F) (Unpaired t test, *t* = 9.811, *n* = 8 per group). (H) Complementary sequences between miR-152-3p and the 3′UTR of Wnt10b. (I) relative luciferase activities of the Wnt10b-wild type (WT) + negative control (NC) group, Wnt10b-WT + miR-152-3p group, Wnt10b-MUT + NC group, and Wnt10b-MUT + miR-152-3p group. (one-way ANOVA, *F* (3, 8) = 43.43, *p* < 0.001. Tukey's post hoc test. *n* = 3 per group). (J) Western blot analysis of the expression of WNT10b and β-CATENIN in the NSCs after LV-wnt10b (LV-NC) and miR-152-3p (miR-NC) treatment. (K) Quantification of WNT10b and β-CATENIN expression in (J) (two-way ANOVA, *F* (4, 30) = 113.0, *p* < 0.001. Tukey's post hoc test. *n* = 4 per group). (L) Neurosphere assay showed the secondary neurospheres size under LV-wnt10b (LV-NC) and miR-152-3p (miR-NC) administration. (M) Quantitative analysis of average neurosphere diameter in (L) (one-way ANOVA, *F* (4, 45) = 29.61, *p* < 0.001. Tukey's post hoc test. *n* = 10 per group). (N) Neural differentiation assay showed the different rate of NSCs under LV-wnt10b (LV-NC) and miR-152-3p (miR-NC) administration. (O) Quantitative analysis of neural different proportion in (N) (one-way ANOVA, *F* (4, 20) = 47.00, *p* < 0.001. Tukey's post hoc test. *n* = 10 per group). Data are presented as mean ± SD, NS, no significant difference, *P < 0.05, **P < 0.01, ***P < 0.001.

To explore the mechanisms and the targets of microglia-derived miR-152-3p on hippocampal neurogenesis following SCI, the NSCs from the hippocampus of mice after SCI or sham surgery using flow cytometry (Figure S8 A-C) and obtained the gene expression profiles of NSCs from both injured and control groups using mRNA sequencing. The gene profiles of the isolated NSCs from the sham our SCI mice were shown by volcano plot (Figure 6A). The top 20 different expressed genes were shown by heatmap (Figure 6B). To identify the targets of miR-152-3p in hippocampal NSCs, we utilized online tools for miRNA target prediction in conjunction with sequencing results that showed downregulated mRNA expression 34, 35. Four potential regulatory sites were identified as miR-152-3p target genes, which are *Wnt10b, Col2a1, Txnip, and Stx3* (Figure 6C-D). Using qRT-PCR, the downregulation of these mRNAs in the hippocampus of SCI mice was confirmed. However, the study found that only the expression of *Wnt10b* could be downregulated by intervention with SCI-EVs (Figure 6E). The immunofluorescence staining results showed decreased expression of wnt10b in hippocampal NSCs following SCI, suggesting that the expression of WNT10b can be regulated by EVs derived from SCI tissue (Figure 6F-G). The luciferase reporter experiment was used to determine if the Wnt10b-3′UTR is a direct target for miR-152-3p, it was found that miR-152-3p mimics dramatically decreased the luciferase activity of the wild type (WT), while the luciferase activity of the mutant type (Mut) of 3′UTR reporter constructs was not altered (Figure 6H-I). Lentivirus for wnt10b overexpression and miR-152-3p mimics were constructed to intervene in NSCs cultured *in vitro*. Assessment of WNT pathway activation via β-CATENIN showed that miR-152-3p inhibited Wnt10b expression and pathway activity. Conversely, Wnt10b overexpression increased β-CATENIN expression, which could be suppressed by miR-152-3p. (Figure 6J-K). Using neurospheres formation assays and neural differentiation assay, we validated the role of miR-152-3p/WNT10b in the proliferation and neurogenesis of NSCs. The results consistently demonstrated that the activation of WNT10b can promote the proliferation and differentiation of NSCs, while the intervention of miR-152-3p can inhibit the effects of WNT10b (Figure 6L-O). These results indicated that *Wnt10b* in hippocampal NSCs is a key target of EVs-derived miR-152-3p from SCI-activated microglia.

### Activation of the WNT pathway in the hippocampus effectively alleviated the suppression of hippocampal neurogenesis and cognitive dysfunction induced by SCI

**Figure 7:**
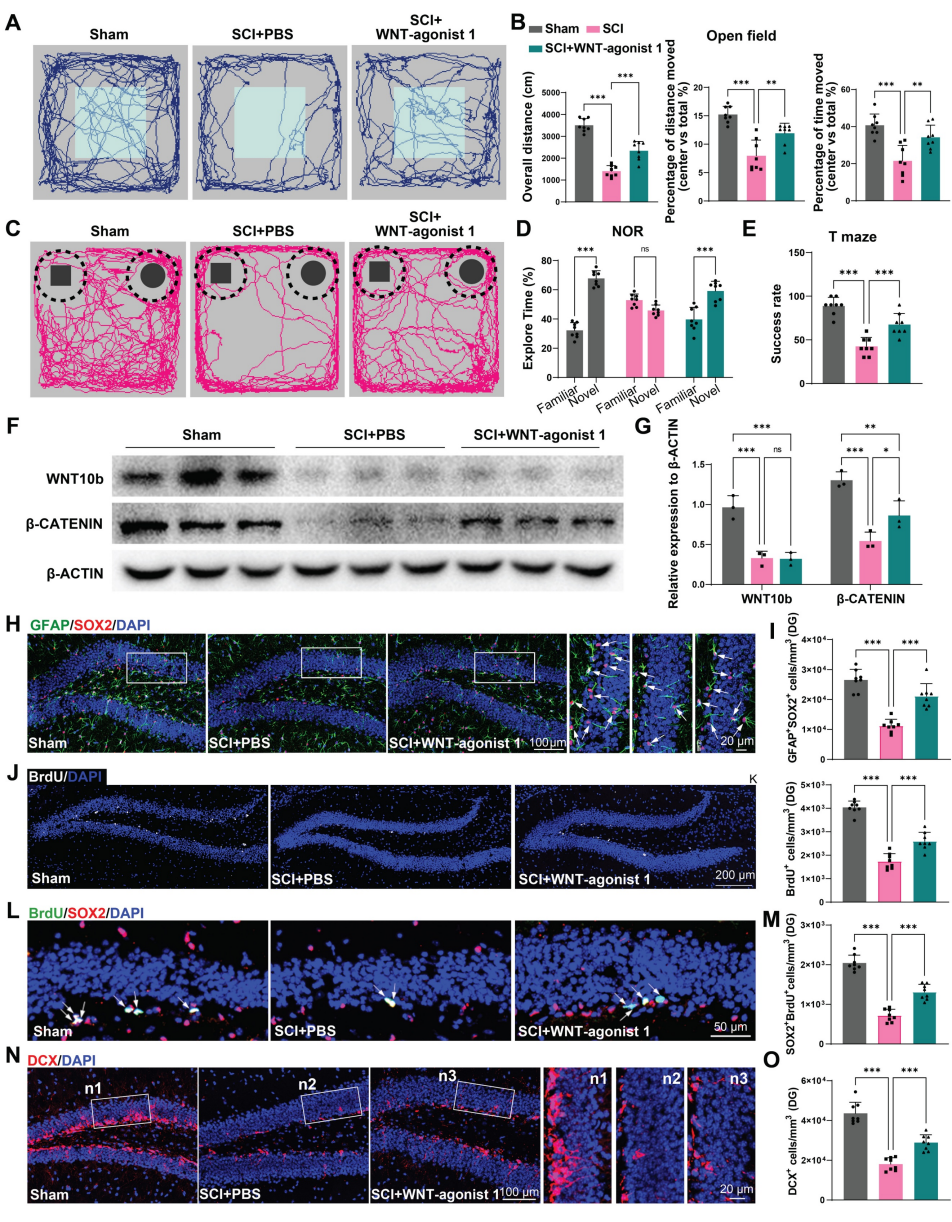
** Activation of the WNT pathway in the hippocampus effectively alleviated the suppression of hippocampal neurogenesis and cognitive dysfunction induced by SCI.** (A) Trajectory plots illustrating mice movement during the open field test. (B) Quantification mice total move distance (one-way ANOVA, *F* (2, 21) = 77.88. *p* = 0.0031 Tukey's post hoc test. *n* = 8 per group), percentage of move distance in center (one-way ANOVA, *F* (2, 21) = 24.69, *p* = 0.0109. Tukey's post hoc test. *n* = 8 per group) and percentage of time spent in the central region (one-way ANOVA, *F* (2, 21) = 15.37, *p* < 0.001. Tukey's post hoc test. *n* = 8 per group). (C) Trajectory plots illustrating mice movement during the novel objection reorganization test. (D) Proportion of time spent with the novel or familiar objections (one-way ANOVA, *F* (2, 42) = 56.17, *p* < 0.001. Tukey's post hoc test. *n* = 8 per group). (E) Quantification of success rate of spontaneous alternation of mice (one-way ANOVA, *F* (2, 21) = 34.80, *p* < 0.001. Tukey's post hoc test. *n* = 8 per group). (F) Western blot analysis of the expression of WNT10b and β-CATENIN in the hippocampal NSCs after WNT-agonist treatment. (G) Quantification of WNT10b and β-CATENIN expression in (F) (one-way ANOVA, *F* (2, 9) = 24.92, *p* < 0.001. Tukey's post hoc test. *n* = 3 per group). (H) Representative immunofluorescent stains of the radial glia-like cells (GFAP, green fluoresce, SOX2, Red fluoresces) images of the hippocampus at 8 weeks post-injury in each group. Scale bar, 100 μm. (I) Quantification of GFAP^+^SOX2^+^ cells of sham and SCI groups in (H) (one-way ANOVA, *F* (2, 21) = 41.47, *p* < 0.001. Tukey's post hoc test. *n* = 8 per group). (J) Representative immunofluorescent stains of the BrdU^+^ cells (BrdU, white fluoresces) images of the hippocampus at 8 weeks post-injury in each group. Scale bar, 200 μm. (K) Quantification of BrdU^+^ cells of sham and SCI groups in (J) (one-way ANOVA, *F* (2, 21) = 99.74, *p* < 0.001. Tukey's post hoc test. *n* = 8 per group). (L) Representative immunofluorescent stains of the SOX2^+^BrdU^+^ cells (SOX2, Red fluoresces, BrdU, green fluoresces) images of the hippocampus at 8 weeks post-injury in each group. Scale bar, 50 μm. (M) Quantification of SOX2^+^BrdU^+^ cells of sham and SCI groups in (L) (one-way ANOVA, *F* (2, 21) = 102.8, *p* < 0.001. Tukey's post hoc test. *n* = 8 per group). (N) Representative immunofluorescent stains of the neuroblast (DCX, Red fluoresces) images of the hippocampus at 8 weeks post-injury in each group. Scale bar, 100 μm. (O) Quantification of DCX^+^ cells of sham and SCI groups in (N) (one-way ANOVA, *F* (2, 21) = 70.09, *p* < 0.001. Tukey's post hoc test. *n* = 8 per group). Data are presented as mean ± SD, NS, no significant difference, *P < 0.05, **P < 0.01, ***P < 0.001.

To confirm the role of WNT pathway in neurogenesis after SCI, using stereotactic techniques, WNT-agonist 1 was directly injected into the hippocampal tissue of mice. Cognitive function was analysis after WNT-agonist injection. It was observed that the mice treated with AAV-miR152-IN exhibited no significant improvement in motor function recovery (Figure S9A). However, these mice treated with WNT-agonist 1 exhibited ameliorated depressive-like and anxiety alterations (Figure 7A-B) and improved recognition and learning memory capabilities (Figure 7C-E). Western blot was employed to confirm the effects of the WNT-agonist 1, and the results indicated that the WNT-agonist 1 could upregulate the expression of β-CATENIN without affecting the expression of WNT10b (Figure 7F-G). And a significant increase in BrdU^+^ and SOX2^+^GFAP^+^ RGL cells was observed in the DG of the WNT-agonist 1 treated mice (Figure 7H-K). Furthermore, a marked increase in SOX2^+^BrdU^+^ cells in the SGZ of SCI mice suggested a curtailed self-renewal capacity of NSCs in the WNT-agonist 1 treated mice (Figure 7L-M). Consistently, an increase in DCX^+^ immature neurons was noted, indicative of suppressed hippocampal neurogenesis after WNT-agonist 1 administration (Figure 7N-O). These results indicated that WNT-agonist 1 ameliorated neurogenesis in the mouse hippocampus, resulting in cognitive reparation post-SCI.

## Discussion

**Figure 8:**
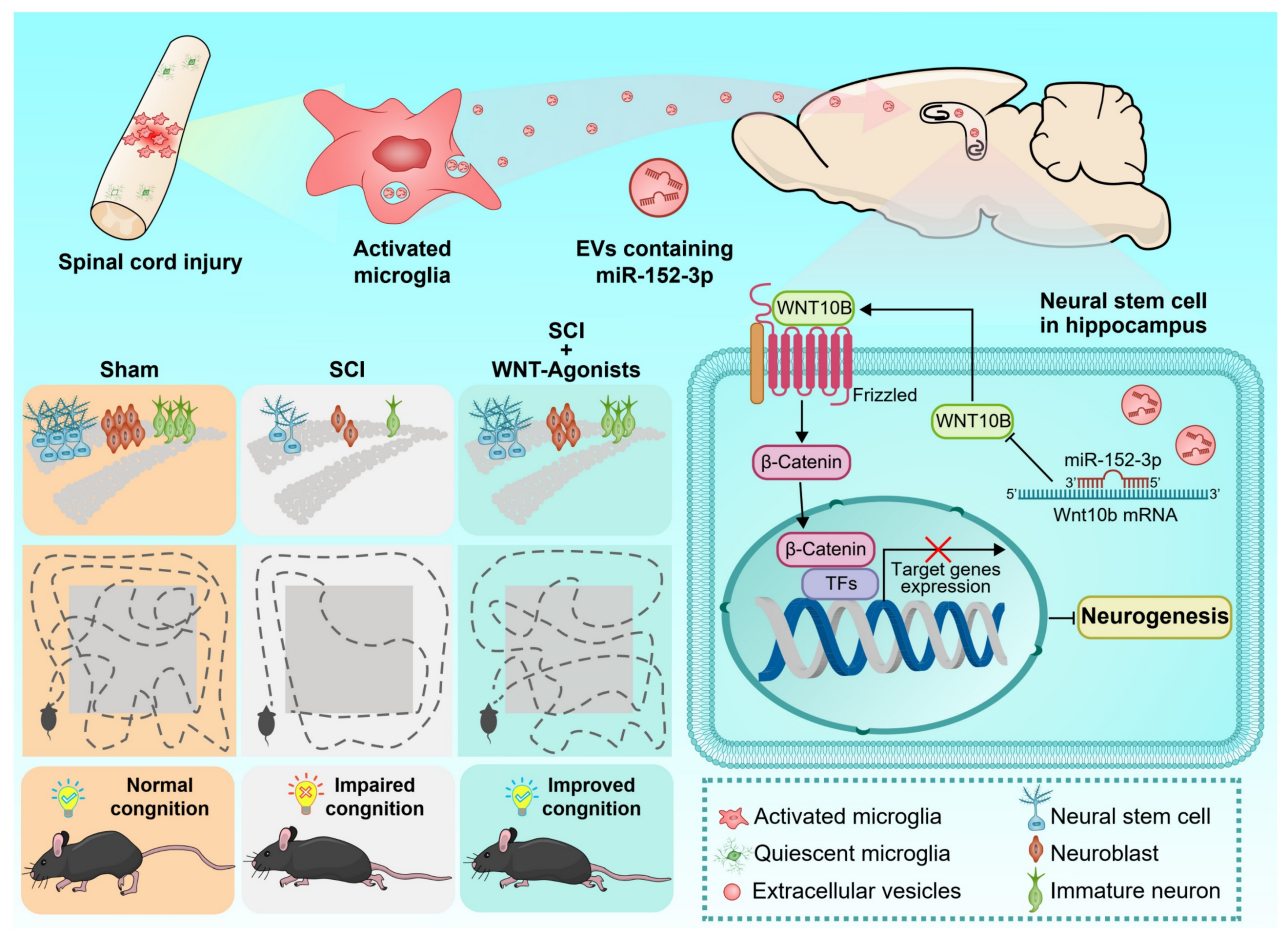
** Graphic abstract.** Following spinal cord injury (SCI), activated microglia in the lesioned area release abundant extracellular vesicles (EVs) containing miR-152-3p. These microglia-derived EVs systemically transport miR-152-3p to hippocampal neural stem cells (NSCs), which subsequently disrupt hippocampal neurogenesis and cognitive function through targeting the WNT10b/β-catenin signaling axis. Mechanistically, the miR-152-3p/WNT10b/β-catenin regulatory circuit constitutes a key pathological mediator in SCI-induced cognitive impairment. Importantly, pharmacological activation of the WNT pathway in hippocampal NSCs post-SCI effectively rescues neurogenesis deficits and improves cognitive behavioral outcomes in murine models. This study elucidates a novel pathological pathway linking remote spinal cord lesions to hippocampal dysfunction, revealing potential therapeutic targets for SCI-related cognitive deficits.

In this investigation, we initially characterized the EV-mediated communication between the injured spinal cord and the hippocampus, identifying activated microglia at the lesion site as the principal cells accountable for EV release. Our findings further elucidate that the miR-152-3p/WNT10b pathway serves as a critical regulatory hub in this pathological mechanism. The stimulation of the WNT pathway in hippocampal NSCs post SCI markedly improves cognitive and behavioral outcomes in mice with SCI. Our research establishes a clear link between spinal cord pathology and hippocampal function, illuminating the intrinsic mechanisms and potential therapeutic targets for cognitive impairments related to SCI (Figure 8).

Adult NSCs or precursors located in the subventricular zone and the DG can differentiate and proliferate into new neurons 36, 37. Research has suggested that the suppression of neurogenesis in hippocampal NSCs following SCI may be a possible explanation for the observed decline in cognitive abilities, as the hippocampus is associated with memory storage and processing 38, 39. Adult hippocampal neurogenesis, which has a direct impact on cognitive function, has been well-documented in mammalian systems since the mid-1990s, primarily occurring in the subventricular zone and the dentate gyrus (DG) of the hippocampus 27, 28. Therefore, investigating how SCI affects hippocampal NSCs and subsequently leads to cognitive dysfunction is of great importance. Studies have revealed the potential mechanisms which may contribute to the impaired hippocampal neurogenesis. Junfang Wu and others have long been concerned about the issue of neuroinflammation after spinal cord injury. They found that the local inflammation level caused by microglia in the SCI area can affect the inflammation level in the cortex of the mouse brain, leading to depression and cognitive impairment in mice 22. As the extracellular vesicles begin to serve as widespread intercellular communication mechanism for communication between tissues and organs 40, Wu's subsequent research also noted that spinal cord injury can affect the changes in the miRNA components of plasma exosomes, which may be related to the exosomes released by local cells at the injury site 41. Therefore, in this study, we not only focused on the changes in hippocampal neurogenesis caused by spinal cord injury, but also investigated the role and molecular mechanism of extracellular vesicles released by microglia in the spinal cord injury area in the spinal cord-brain interaction. We discovered a communication bridge among spinal microglia, extracellular vesicles and hippocampal neural stem cells through the miR-152-3p/Wnt10b mechanism, further clarifying the pathological mechanism basis of cognitive dysfunction caused by SCI.

Given that spinal cord injury (SCI) typically induces severe motor dysfunction and most murine behavioral assays (e.g., forced swim test, tail suspension test, Morris water maze) critically depend on intact locomotor function, available behavioral paradigms for SCI evaluation remain highly limited. To eliminate confounding effects of motor impairment, BMS scores were assessed in miR-152-3p-inhibited and Wnt agonist 1-treated SCI mice, confirming that neither intervention affected motor functional recovery. Given the compromised physical condition of SCI mice, simplified behavioral tests including open field, novel object recognition, and T-maze assays were employed. Conventionally, the open-field test is a behavioral test method for anxiety 42. However, mice with depression can also show certain changes in behavioral patterns during the open-field test. For example, a decrease in the total activity of the animal during the experiment may indicate depressive behavior and some studies have found that depressed animals may also show avoidance of the central area and a decrease in the willingness to move 43-46. In this research, the results revealed the SCI mice showed reduced exploratory motivation and peripheral preference in open field (analyzed via standardized metrics: movement time ratio; central/total movement distance ratio). This result suggests that mice with spinal cord injury have anxiety or depression like emotional disorders. There is a bidirectional interaction mechanism between depressive symptoms and cognitive function, which means depression-like behaviors can lead to cognitive impairment, and a decline in cognitive level can also exacerbate depression-like phenotypes 47-49. Based on the above theories, we further systematically evaluated the cognitive function of mice through the novel object recognition (NOR) test and the T-maze test. Novel object recognition and T-maze tests further demonstrated impaired recognition memory and spatial memory in SCI mice 50, 51. Notably, suppressing miR-152-3p in spinal microglia or activating Wnt signaling in hippocampal dentate gyrus (DG) effectively alleviated these cognitive deficits, providing critical mechanistic insights into spinal cord-brain communication after SCI. However, there are still quite a number of behavioral experiments for detecting cognitive levels that are closely related to hippocampal neurogenesis, such as the novel location recognition experiment and the reversal learning experiment, which were not conducted in our study 52-54. With the further in-depth exploration of the functional changes in the brain regions of mice with spinal cord injury, conducting more targeted behavioral tests on mice with spinal cord injury is of great significance for us to understand the potential risks of cognitive impairment in neural function caused by spinal cord injury.

EVs can transfer a variety of bioactive components, including miRNA, mRNA, DNA, and proteins, exerting effects on recipient cells 29-31. Among these, miRNAs loaded onto EVs are considered important substrates for their functional effects. miRNAs are a class of single-stranded non-coding RNAs that induce RNA degradation by directly binding to the 3'-UTR (untranslated region) of target gene mRNAs 55, 56. Extensive research has revealed pronounced changes in the miRNA expression landscape within animal models of SCI, characterized by the upregulation or downregulation of specific miRNAs 57-60. These differentially expressed miRNAs post-SCI are potential key targets for diagnostics, prognostication, elucidating molecular mechanisms, and therapeutic intervention 61. Notably, miRNA-219 and miRNA-146a have been demonstrated to foster neural functional restoration by mitigating apoptosis and the reactive astrocyte response to injury, acting as protective factors in the context of SCI 62. In contrast, miRNA-411 and miRNA-99a are associated with an intensified inflammatory response to injury, indicating their role as detrimental factors following SCI 63, 64. And numerous studies have also suggested that the biological functions of miRNAs may be related to hippocampal NSCs which could potentially serve as therapeutic targets for the treatment of hippocampal NSCs 65-68. However, there is currently no studies on the relationship between hippocampal NSCs and cognitive dysfunction following SCI. To clarify the mechanisms leading to cognitive dysfunction after SCI, we analyzed single-cell public databases from SCI mice and found that the secretion of EVs from various cells increased after SCI, such as microglia, macrophages, and oligodendrocyte precursor cells. Using immunofluorescent co-staining of microglia marker (IBA-1) and EVs surface marker (CD63), it was confirmed that microglia are the richest source of exosome secretion after SCI. To explore the mechanisms by which microglial EVs exert their biological effects, we performed microRNA-seq on purified Sham-EVs and SCI-EVs and found that miR-152-3p is a key factor in the biological functions of SCI-EVs. In previous studies, miR-152-3p has been considered a tumor suppressor in various types of cancer, including breast, prostate, colorectal, and glioma 69-72. To date, there have been no reports on the association between miR-152-3p and cognitive dysfunction following SCI. In this study, we have uncovered and substantiated a novel association between cognitive dysfunction following SCI which regulated by miR-152-3p, a microRNA derived from microglia within the spinal tissue. Our findings suggested that the dysregulation of miR-152-3p in spinal microglia play a critical role in the pathogenesis of cognitive impairments observed post-SCI. This discovery not only sheds light on the underlying molecular mechanisms but also offers potential avenues for therapeutic intervention, as targeting miR-152-3p could mitigate cognitive decline in individuals with SCI.

WNTs are a series of glycoproteins which act as a ligand to bind the N-terminal cysteine-rich domain of the Frizzled family receptors 73. WNT signaling governs multiple aspects of neural development including neurulation, pattern formation, and neurogenesis 74. It has been reported that WNT/β-CATENIN pathway was associated with enhanced neurogenesis upon immune-mediated neuroinflammation 75. Another research proved that WNT/β-CATENIN signaling activity decreased with aging, which makes it difficult for new-born neurons in the hippocampus to mature, while enhancing WNT/β-CATENIN signaling promoted neurons maturation with increased dendritogenesis 76. In some neurodegeneration diseases such as Alzheimer's disease (AD), WNT/β-CATENIN signaling could benefit neuronal maturation and synapse formation, relieving the cognitive dysfunction caused by AD 77-79. Exogenous WNT protein is also considered to be an effective therapy for improving cognitive dysfunction. Studies showed that bone marrow mesenchymal stem cells (BMSCs) derived WNT3a could promote hippocampal neurogenesis and facilitate neurocognitive outcomes through activating β-CATENIN after traumatic brain injury 80. However, whether WNT pathway was also involved in SCI-induced hippocampal neurogenesis damage was rarely reported. In this study, we utilized a comprehensive approach to examine both the miRNA expression profile of EVs derived from injured spinal cord tissue and the mRNA expression profile of hippocampal NSCs. We identified WNT10b as a target gene regulated by miR-152-3p and confirmed that the activation of WNT pathway in hippocampal NSCs could rescue the cognitive deficit following SCI.

In conclusion, this research elucidates a previously unidentified EVs-mediated crosstalk between the brain and spinal cord, demonstrating that microglia-originated EVs following SCI are replete with miR-152-3p. Upon uptake by hippocampal NSCs, these vesicles impede the neurogenic differentiation of the stem cells, consequently culminating in post-SCI cognitive deficits.

## Method and Materials

### Animals

Studies have also shown that female mice with spinal cord injury are less likely to exhibit cognitive function disorders, which may be related to the estrogen levels in mice 81-83. Therefore, to avoid the interference of gender-related hormonal fluctuations on the research results, we chose male mice as the research model. In this experiment, 8-week-old male C57BL/6J (wild type, WT) mice were purchased from Hunan SJA Laboratory Animal Co., Ltd. C57BL/Cx3cr1-CreERT2 were procured from Shanghai Model Organisms Center, Inc. The construction of the spinal cord injury model, animal housing and care, animal perfusion, and specimen acquisition were all carried out in the Department of Laboratory Animals, Central South University. Cryosections, immunofluorescence staining, quantitative real-time PCR (qRT-PCR), Western blot (WB), and other procedures were conducted in the Key Laboratory of Organ Injury, Aging and Regenerative Medicine of Hunan Province. All procedures were conformed with the Helsinki Declaration and approved by the Ethics Committee of Central South University (CSU-2024-0008).

A total of 181 male mice were used for cognitive and IHC analysis. Among the 181 mice, 17 mice were excluded due to severe urinary tract infections or accidental death. 80 mice were randomly assigned to the sham and SCI groups (32 in sham groups, 48 in SCI groups). Among the 80 mice, 20 mice in sham group and 20 SCI mice were used for spinal EVs extracting, hippocampal NSCs isolation and microglia in spinal cord and hippocampus isolation; 24 mice of the sham and SCI groups (n = 4/time point) were scarified for time-point analysis of the extracellular vesicle's marker expression; 16 mice of the sham and SCI groups were used for behavior analysis and immunofluorescence staining. To test the effects of EVs derived from the injured spinal cord on neurogenesis, 24 mice were randomly assigned to the control, Sham-EVs and SCI-EVs groups (n = 8, per group). To test the effects of miR-223-3p and miR-152-3p on neurogenesis, 12 naive mice were randomly assigned to the groups (n = 4, per group). To clarified the role of microglia-derived miR-152-3p in hippocampal neurogenesis, 24 SCI mice were randomly assigned to the Control, AAV-NC and AAV-miR152-IN groups (n = 8 per group). To further evaluate the efficacy of wnt-agonist1 on impaired hippocampal neurogenesis after SCI, 24 mice were randomly assigned to three groups: Sham, SCI + PBS, and SCI + WNT-agonist1 (n = 8 per group). The samples for IHC analysis were collected at 56 days post injury or treatment, unless otherwise specified.

### Spinal cord injury construction

Eight-week-old male C57BL/6J mice, weighing 20-25 g, were acclimated in a barrier environment at Central South University's Department of Laboratory Animals. Anesthetized with pentobarbital sodium (50 mg/kg), the mice underwent dorsal preparation, including shaving, alcohol and povidone-iodine disinfection, and sterile draping. A 3 cm × 1.5 cm area over the T10 vertebrae was cleared of fur for surgery. The surgical site was disinfected, and a 1cm incision was made at the T10 level to expose the spinal cord. A modified Allen's impact method using a 10 g weight dropped from 2.5 cm induced the injury, confirmed by hematoma and limb movements. Post-surgery, the incision was closed with layered sutures and iodine disinfection. Sham-operated mice served as controls. Mice were housed at 4-5 animals per cage with libitum access to food and water. After surgery, mice were administered with penicillin (3,000 IU/mL) in the water for three days and their bladders were manually emptied every two days until urinating function was recovery. The SCI mice received Buprenorphine (0.03 mg/kg/day, subcutaneous injection) to alleviate pain during the first week after surgery.

### miR-152-3p inhibition in microglia

5-week-old Cx3cr1-CreERT2 male mice were received an intravenous injection in the tail vein of the pAAV-MG1.2-DIO-EGFP-Sponge(mmu-miR-152-3p)-WPRE viral construct (100 μL 1×10^12^ vg/mL, OBiO Technology (Shanghai) Corp., Ltd.). Following a 2-week transfection period, mice were administered tamoxifen (75 mg/kg/day, Sigma-Aldrich) for 3 consecutive days to induce Sponge(mmu-miR-152-3p) expression in microglia. SCI was established 1-week post-tamoxifen treatment. The animals were maintained in a thermoregulated environment under a 12-hour light-dark cycle, with free access to food and water. The behavior test and IHC analysis was performed at 8 weeks post SCI.

### Tracing of spinal microglia-derived EVs

To investigate the impact of microglia-derived extracellular vesicles (EVs) on hippocampal neurogenesis and cognitive function, we performed local injections of pAAV-MG1.2-DIO-EGFP-P2A-Cd63-3xFLAG-WPRE (AAV-CD63-EGFP) (0.5 μL 1×10^12^ vg/mL, OBiO Technology (Shanghai) Corp., Ltd.) into the T10 spinal cord segment of 5-week-old Cx3cr1-CreERT2 male mice. Age-matched naive male mice received equivalent PBS injections as negative controls (NC). Following a 2-week transfection period, mice were administered tamoxifen (75 mg/kg/day, Sigma-Aldrich) for 3 consecutive days to induce microglia-specific EV labeling through EGFP expression in spinal microglia. Spinal cord injury (SCI) was established 1week post-tamoxifen treatment. 1 week post-SCI, spinal cord and hippocampal tissues were collected for immunofluorescence analysis to validate both transfection efficiency and hippocampal EV localization.

### Blinding in the study design

During the experimental grouping stage, the randomization of mouse numbering is achieved through independent coding to ensure that experimenters cannot identify the groups by cage numbers or markings. Meanwhile, non-behavioral testing personnel are responsible for the grouping operations and seal and preserve the grouping information, which will only be decrypted during the data analysis stage. In the behavioral testing stage, the operators are completely unaware of the grouping information. All mice undergo open-field tests, T-maze tests, novel object recognition tests, etc. in a standardized environment (with unified time, equipment, and light/noise conditions). The Smart 3.0 video tracking system is preferentially used to automatically record data such as movement distance, minimizing the subjective bias introduced by manual intervention.

### Open field

Before the experiment, mice were individually placed in an undivided open-field arena (40 cm × 40 cm) and allowed to acclimate to the quiet environment for 15-30 min. During the experiment, mice were gently suspended by the tail and lowered into the arena, where they were free to move under low-light conditions for 10 min. Their activity and behavioral characteristics were recorded using video equipment or an automated tracking system. Data were subsequently analyzed using computer software to calculate metrics such as distance moved, time spent, and frequency of exploration in the central area (20 cm × 20 cm). After the experiment, the open field was thoroughly cleaned and disinfected.

### Novel Object Recognition (NOR)

The test is conducted in a 40 cm × 40 cm square field. Cube models and cone molds are used as the familiar and novel objects respectively, and the objects are placed 8 cm away from the wall to ensure that the mice can smoothly pass through the gap between the objects and the wall. The experimental protocol is bifurcated into an acclimation phase and a testing phase: 1. In the acclimation phase, mice are introduced to the experimental apparatus one day before the main experiment to acclimate them to the surroundings; 2. During the testing phase, the mice, now in a familiar setting, are initially presented with two identical objects for a 10 min exploration period. One of these objects is then replaced with a novel one, and the mice are given an additional 10 min to explore. By meticulously observing and documenting the mice's engagement with the new object, we assess their recognition of the novel item, which serves as an indicator of their cognitive capacity.

### Spontaneous alteration on T-maze

Gently pet the mice for 1-2 min daily for 5 to 7 consecutive days to familiarize them with human contact and reduce stress. Place the mouse in the stem arm of the T-maze. Open the gate to allow the mouse to exit the stem and enter one of the goal arms (all four paws must be inside the arm). After the mouse enters a goal arm, return it to the stem arm and confine it there for a brief period (start with a 5 s count). Repeat this process 9 times, recording the number of entries into each arm. Ideally, the mouse should alternate choosing between the two goal arms with each trial. This alternation is an indicator of the mouse's ability to remember its previous choice and make a new one. The experimental result is expressed as the number of alternations divided by the total number of choices made during the same experimental interval (10 trials in total).

### Cell culture

Eight-week-old C57BL/6J mice were humanely euthanized via cervical dislocation. Subsequently, the brains were carefully extracted, and the meninges were meticulously removed. Utilizing a dissecting microscope, the hippocampal region from the ventral aspect of the cortical hemispheres was meticulously dissected and transferred to culture medium containing Accutase enzyme (Invitrogen, US) for a 25 min enzymatic digestion. Post-digestion, the Accutase was carefully aspirated, and the hippocampal tissue was gently triturated to achieve a uniform cell suspension, with attention to prevent bubble formation. The resulting hippocampal neural stem cell suspension was collected and centrifuged to eliminate the supernatant. The cell pellet was then resuspended and processed with 22% Percoll (Invitrogen, US) to purge myelin contaminants. Finally, the cells were cultured in pre-warmed (37 ℃) NSC medium, a proprietary blend consisting of neurobasal-A (Invitrogen, US), 0.24% GlutaMAX supplement (Invitrogen, US), 4% B27 supplement devoid of vitamin A (Invitrogen, US), and supplemented with 20 ng/mL EGF (Sigma-Aldrich, US) and 20 ng/mL bFGF (Sigma-Aldrich, US). After 7 days of continuous culture, the neurosphere clusters formed by proliferation were collected. A double-labeling strategy with SOX2 and NESTIN was employed, and the neurospheres were specifically identified by immunofluorescence technique.

### Extracellular vesicles (EVs) extraction

Mice from both the sham surgery and spinal cord injury (SCI) groups were sacrificed on 14 days post-surgery. Segments of spinal cord tissue, approximately 0.6 cm surrounding the lesion site, were meticulously excised and dissected into fragments. These fragments were immersed in cell culture medium and treated with a tissue dissociation enzyme cocktail (0.1% Collagenase A (Roche, Switzerland) + 0.1% Dispase (Sigma-Aldrich, US) + 0.05% DNase (Sigma-Aldrich, US)) to promote the release of extracellular vesicles. To terminate the enzymatic reaction, the samples were diluted 1:1 with PBS containing 2 mmol/L EDTA (Invitrogen, US). The spinal cord tissue culture medium, enriched with extracellular vesicles, was collected for subsequent experimental procedures. The supernatant was initially centrifuged at 4 ℃ and 300 × g for 15 min to pellet any residual cells, after which the supernatant was carefully retrieved, leaving behind any floating cellular debris. The supernatant was then subjected to a second centrifugation at 3,000 × g for 30 min to sediment potential remaining fragments. The supernatant was subsequently filtered through a 0.22 μm filter using a 50 mL syringe, ensuring the removal of any particulate matter. Finally, the filtrate was ultracentrifuged at 120,000 × g for 3 h to concentrate the EVs, which were then collected from the pellet at the bottom of the centrifuge tube after the supernatant was discarded. This meticulous process ensured the acquisition of pure exosome suspensions from the spinal cord tissue of both sham and SCI groups, ready for further analysis and application.

### Immunofluorescence

After anesthetization, the mice were perfused with normal saline and 4% paraformaldehyde via the left ventricle. The brain and T6-T12 spinal cord segment were harvested. After dehydration with 20% and 30% sucrose solutions, the tissues were embedded in optimum cutting temperature compound (Sakura, Torrance, CA, USA) for frozen sectioning. Both the brain and spinal cord tissues were sectioned into slices with 30 μm thickness. Multiplex immunofluorescence staining was performed using Color Multiple Fluorescence Kit (AiFang Biological) according to the manufacturer's instructions. Briefly, delineate the sections on the slide using an immunohistochemistry pen and then immerse them in 100 μL of PBST solution with 0.1% Triton-X100 for 20 min to achieve permeabilization. Subsequently, remove the permeabilization fluid and overlay the sections with 100 μL of a 3% BSA solution for 30 min to quench non-specific interactions. Post-blocking, rinse the sections thrice with PBS. Concoct the primary antibody solution (**Table S1**) in accordance with the volume of the sample and apply it to the sections, followed by an incubation period overnight at 4 °C. On the subsequent day, retrieve the sections and equilibrate them at room temperature for 20 min, then replenish the primary antibody and cleanse the sections with 0.1% PBST, repeating this process twice for 20 min each, complemented by PBS washes for 5 min each time. Formulate the secondary antibody solution based on the sample volume and delicately administer it to the rinsed sections, followed by a 2 h incubation in the dark at room temperature. Once the incubation is complete, perform washes on the secondary antibody as per the primary antibody protocol. Meticulously dry the specimens, apply DAPI staining solution with an anti-fade reagent, mount the slide, and document the results using an APTOME 2.0 fluorescence microscope from Zeiss.

For quantification of cells expressing stage-specific markers, 1 in 15 serial sections starting at beginning of hippocampus (relative to bregma, -1.5 mm) to the end of hippocampus (relative to bregma, -3.0 mm) were used. Quantification of BrdU^+^ cells and cells labeling with SOX2, GFAP, DCX in the region of interest (ROI) of granule layer were performed using APTOME 2.0 fluorescence microscope (Zeiss) with ZEN software in at least 3 sections containing the dentate gyrus from the same animal. All images captured correspond to 30 μm z-stacks and were acquired following protocols optimized for stereological quantification and quantitative image analysis 84. Results were expressed as the number of positive cells per mm^3^.

### BrdU labeling

Three days before tissue collection, BrdU (dissolved in 0.9% sterile normal saline) was intraperitoneally injected daily at a dose of 50 mg/kg body weight for three consecutive days. Sample analysis was performed on the first day after the last injection. Briefly, animals were transcardially perfused with 4% paraformaldehyde (PFA), after which brain tissues were post-fixed in the same fixative for 24 h at 4 °C and cryoprotected in 30% sucrose solution; 30 μm-thick coronal sections were cut using a cryostat (Leica CM1950). BrdU^+^ cells in the granule layer were counted using as previous mentioned 84.

### Flow cytometry

In this research, we utilized fluorescence-activated cell sorting (FACS) to meticulously isolate hippocampal NSCs and microglia from the injured spinal cord of mice. The process commenced with the rapid cervical dislocation of the mice to ensure humane euthanasia, followed by the precise dissection of hippocampal tissues and the injured spinal cord segments. Tissue blocks were then transformed into a suspension of single cells through enzymatic digestion at 37 °C, employing a mixture of 0.25% Collagenase A, 0.1% Dispase, 0.05% DNase, 10 mM HEPES, and Hanks' balanced salt solution. Post-digestion, the cells were rinsed with FACS buffer (PBS and 1% FBS), and the anti-mouse CD16/CD32 antibody (Fc Receptor Blocking Solution, BD, US) was added to the cell suspension at a 1:50 dilution, followed by a 15 min incubation at 4 °C to block Fc receptors. The cells were then resuspended in staining buffer (BD, US) and incubated with a panel of antibodies (panel of NSCs isolation: CD45, CD133, GLAST, panel for microglia isolation: DAPI, CD45, CD11b) for 30 minutes at 4°C (as detailed in Table S1). After staining, cells were washed and resuspended in PBS supplemented with 2 mM EDTA (Thermo Fisher Scientific, USA) and 1 μg/mL 4',6-diamidino-2-phenylindole (DAPI, BD, US) to exclude dead cells. The cell sorting was performed on a FACS Aria II SORP cell sorter (BD Biosciences, US). The cells based on the DAPI⁻/CD45^low^/CD11b⁺ marker combination are identified as microglia and screened by flow sorting technology, while the cells conforming to the DAPI⁻/CD45⁻/GLAST⁺/CD133⁺ marker system are specifically recognized as neural stem cells and subjected to targeted sorting. The resulting data were meticulously analyzed using FlowJo software (TreeStar, US).

### qRT-PCR

Total RNA was extracted from EVs (isolated EVs from sham or injured spinal cord), injured spinal cord (time point post spinal cord injury as indicated) and microglia sorted from the spinal cord and hippocampal tissues at 14 days post injury using TRIzol reagent (Invitrogen, US). Subsequently, cDNA was reverse transcribed following the manufacturer's instructions with the PrimeScript™ RT Reagent kit (Promega, US). Additionally, miRNA was reverse transcribed using the miRNA First Strand cDNA Synthesis kit (Tailing Reaction, Sangon Biotech, CN). Pri-miRNAs were synthesized into cDNA with the miRNA 1st Strand cDNA Synthesis Kit (by stem-loop, Sangon Biotech, CN). Quantitative measurement of mRNA and miRNA was conducted using the GoTaq qPCR Master Mix kit (Promega, US) and a quantitative PCR system (ABI, US). To assess the relative expression levels of mRNA or miRNA, the 2^-ΔΔCT method was employed with GAPDH and U6 serving as internal references. The primers utilized for qRT-PCR are listed in **Table S2**.

### Western blot

Proteins were extracted from EVs, spinal cord (time point as indicated) and hippocampal samples (56 days post injury) utilizing RIPA lysis buffer (Beyotime, CN). Protein quantification was accomplished with a BCA protein assay kit (Multisciences, CN). Equivalent aliquots of protein were resolved via 10% SDS-PAGE and subsequently transferred to PVDF membranes. These membranes were blocked with 5% skim milk at room temperature for 2 h to mitigate non-specific interactions. Post-blocking, the membranes were incubated with primary antibodies overnight. Subsequent to the primary antibody treatment, the membranes underwent three washes with TBST containing 0.1% Tween 20, followed by a 90 min incubation with HRP-conjugated secondary antibodies at room temperature. After incubation, the membranes were rinsed thrice with TBST for 10 min each. The detection of immunoreactive bands was achieved using an enhanced chemiluminescence (ECL) substrate. The antibodies utilized for Western blot are listed in **Table S1.**

### microRNA-seq

The EVs were isolated from the spinal cord from the sham or SCI mice at 14 days post injury. After Total RNA from EVs was extracted using the exoRNeasy Serum/Plasma Midi Kit (Qiagen, US) and assessed for quality using a NanoDrop ND-1000 and Agilent 2100 Bioanalyzer. Qualified RNA was subjected to library construction with a small RNA sample prep kit, leveraging the specific termini of small RNAs for efficient ligation. cDNA was synthesized post-ligation and amplified by PCR to obtain target DNA fragments. The cDNA library was purified via gel electrophoresis and its integrity was confirmed using the Agilent 2100 Bioanalyzer. Pooled libraries were sequenced on the Hiseq/Miseq platform to explore the genomic profile. The miRNA sequencing was completed at Wuhan Seqhealth Co., Ltd., China.

### Neuroshpere assay

Transfer the neurospheres-containing medium from the culture plate to a 15 mL centrifuge tube and centrifuge at 300 × g for 5 min. Aspirate the supernatant, and gently resuspend the neurospheres in 1 mL of pre-warmed 0.05% trypsin-EDTA, then incubate at room temperature for 3 min to facilitate dissociation. Following this, add an equal volume of DNaseI-containing trypsin inhibitor and mix well to halt the enzymatic reaction. After another centrifugation at 300 × g for 5 min, discard the supernatant and resuspend the cells in 1 mL of growth medium. Use a pipette to triturate the cell suspension gently about 10 times to ensure complete dissociation of the neurospheress. Withdraw 10 μL of the cell suspension, mix it with an equal volume of trypan blue, and determine the viable cell count using a hemocytometer. Seed the cells at a density of 1 × 10^4^ cells/cm^2^ into appropriately sized culture plates or flasks. Culture the cells in a 37 °C incubator with 5% CO_2_ to allow for the formation of secondary neurospheres.

### Luciferase reporter assay

Wnt10b plasmid, mimics of miR-152-3p, and miR-152-3p negative control (NC) were constructed by Hanbio Biology (China, Shanghai). The wild-type Wnt10b containing miR-152-3p binding site and the binding sequence of 3'UTR and mutation were cloned into a psi-check 2 plasmid, which was called Wnt10b-WT and Wnt10b-MUT respectively. The neural stem cells were co-transfected with luciferase reporter gene constructs (Wnt10b-WT or Wnt10b-MUT) and miRNAs (NC or miR-152-3p). Two days after transfection, the double luciferase reporter gene detection kit was used to detect the fluorescence intensity ratio of NSCs in each group. The sequence of miR-152-3p is 5'GGUUCAAGACAGUACGUGACU3'; the sequence of miR-152-3p-NC is 5'UCACAACCUCCUGAAAGAGUAGA3'.

### Neural differentiation assay

In experiments aimed at inducing neural differentiation of NSCs, isolated NSCs are seeded onto coverslips precoated with Poly-D-lysine and laminin (Sigma, US) in 24-well plates. The cells are maintained in a differentiation medium composed of Neurobasal-A medium supplemented with 2% B27, 1% fetal bovine serum (GIBCO, Life Technologies, US), and 1 μM retinoic acid. The medium is refreshed every 48 h for a duration of approximately 5 days to encourage the differentiation process. To assess the differentiation outcome, immunofluorescence staining is performed using a β-tubulin (TUJ1) antibody to visualize and quantify the proportion of neurons that have successfully differentiated.

### Stereotaxic injection

Mice were anesthetized with an intraperitoneal injection of 0.3% pentobarbital sodium and subsequently positioned on a stereotactic frame in a horizontal orientation, with careful adjustment to ensure axial symmetry and even skull positioning. The surgical site was prepared by shaving with an electric razor and disinfected using 75% ethanol. A 1.5 cm midline incision was made, and the subcutaneous tissues were gently retracted to reveal the skull. The bregma points were identified, and the stereotactic apparatus was aligned accordingly, with the three-dimensional coordinates meticulously noted. Utilizing a stereotaxic mouse brain atlas, the DG region was targeted at the following coordinates: X = ±1.5 mm, Y = -2.0 mm, Z = -2.0 mm. The stereotactic needle was then removed, and a 1 mm diameter hole was drilled at the predetermined X and Y coordinates. Debris was meticulously cleared from the holes using alcohol-soaked cotton. A microsyringe equipped with a flat-tipped needle was secured and positioned perpendicularly to the skull, with the needle advanced to a depth of 2 mm for precise infusion. To evaluate the proliferative effects of EVs and miR-152-3p on hippocampal NSCs, 1 μL of miR-152-3p mimic (10 μg/μL in PBS) or EV suspension (10 μg/μL in PBS) was stereotaxically microinjected bilaterally into the hippocampal dentate gyrus of 8-week-old naive male C57BL/6J mice at the rate of 0.1 uL/min. To evaluate the therapeutic effect of Wnt agonist1 on neurocognitive dysfunction after spinal cord injury, 1μL of Wnt agonist1 (1 μg/μL) was bilaterally injected into the DG of the mouse hippocampus and injection rate being the same as before. Behavioral assessments and immunohistochemical (IHC) analysis of NSC proliferation and neural differentiation markers were subsequently performed at 56 days post-injection, with stereological quantification conducted on 30 μm-thick coronal sections.

### RNA extraction, library preparation and sequencing

Total RNA, isolated from sorted hippocampal NSCs using TRIzol reagent (Ambion, US), underwent DNase I digestion to eliminate genomic DNA contamination. The RNA's quality and integrity were rigorously assessed, and the quantification of qualified RNA was performed using a Qubit3.0 fluorometer. Employing the KC-Digital TM Stranded mRNA Library Prep Kit for Illumina® (Wuhan Seqhealth Co., Ltd., China) in accordance with the manufacturer's protocol, stranded RNA-sequencing libraries were crafted from 2 μg of total RNA. Pre-amplified cDNA was tagged with unique molecular identifiers (UMIs), each consisting of 8 random nucleotides, to correct for biases introduced during PCR amplification and sequencing. The resultant library products, spanning 200 to 500 base pairs, were enriched, quantified, and subjected to sequencing on the DNBSEQ-T7 platform (MGI Tech Co., Ltd., China). Low-quality sequences were discarded, and raw data was refined by removing adapter-derived contaminants with Trimmomatic version 0.36. Subsequently, clean reads were grouped into clusters based on their UMI signatures, and intra-cluster reads were pairwise aligned. Reads with sequence similarity exceeding 95% were consolidated into distinct sub-clusters, from which a consensus sequence was extracted through multiple sequence alignments. Following the removal of duplicates, an in-depth RNA-seq analysis was conducted, shedding light on the underlying transcriptomic profiles.

### Fluorescence* in situ* hybridization

For the detection of miR-152-3p via fluorescence *in situ* hybridization (FISH), a biotinylated miR-152-3p probe (Sangon Biotech, CN) was synthesized. The brain of mice was harvested after perfused with 4 % paraformaldehyde at 14 days post injury. Following gradient alcohol dehydration, the brains were embedded in paraffin, horizontally sectioned at a thickness of 8 μm. For staining, tissue sections were deparaffinized using dimethylbenzene, and rehydrated through a graded ethanol series. Subsequently, the sections were subjected to proteinase K (Ambion, US) treatment and pre-hybridized at 37 °C for 1 h. After discarding the pre-hybridization buffer, the sections were incubated with the biotinylated miR-152-3p probe at 60 °C for an extended period. Post-incubation, the sections underwent a series of washes with 2 × SSC, 1 × SSC, and 0.5 × SSC. A blocking step with a buffer containing 3% BSA in 0.1% PBST was performed for 1 h at room temperature, prior to 1 h incubation with Cy3-conjugated streptavidin (Sigma, US). NSCs was labelled using anti-SOX2 and anti-GFAP primary antibodies, complemented by appropriate secondary antibody staining. Nuclei were counterstained with DAPI. The relative immunofluorescence intensity of miR-152-3p within SOX2^+^GFAP^+^ cells was quantified utilizing ImageJ software.

### 3D-τ-STED/STED-FLIM for EVs detecting

To validate the colocalization of PKH26 and CD63-EGFP in spinal cord-derived EVs, we applied pAAV-MG1.2-DIO-EGFP-P2A-Cd63-xFLAG-WPRE, which could target microglia and make the EVs released by microglia carry the CD63-EGFP fusion protein. We then collected spinal cord tissue from the injury site and extracted EVs. And the collected EVs were labeled with PKH26 and observed by Leica STELLARIS 8 Stimulated emission depletion (STED) microscope. Briefly, the EVs were mixed in the Anti-fluorescence quenching agent (ProLong™ Live Antifade Reagent, Thermal Fish, 1 μg/μL). Subsequently, labeled EVs were imaged with STED microscope using the 100× objective (1.4 NA oil). To improve the signal to noise ratio, STED microscope with Fluorescence Lifetime Imaging (STED-FILM) were performed using the tauSTED mode at a fluorescence lifetime range from 0.5 ns to 8 ns. Pixel size was limited to less than 30 nm. Use ImageJ to calculate the ratio of the number of green fluorescent spots to that of red fluorescent spots in five random fields of view from different samples.

### Statistical analysis

Statistical analyses were conducted using GraphPad Prism version 10. For pairwise comparisons, Student's t-test was applied, and one-way ANOVA was used to evaluate differences across multiple groups. Tukey's multiple comparisons post-test was subsequently implemented for post-hoc analysis. Data are expressed as the mean ± standard deviation (Mean ± SD), with statistical significance defined as *P* < 0.05.

## Supplementary Material

Supplementary figures and tables.
