# Supplementary material for: Extracellular vesicle-mediated spinal cord-brain crosstalk induces hippocampal neurogenesis impairment and cognitive deficits post-spinal cord injury
Source: Theranostics. 2025 Jun 23;15(15):7584–606. doi: 10.7150/thno.110560 (PMC12316029; doi:10.7150/thno.110560)
Supplement: Supplementary file 1 — Supplementary figures and tables. [file thnov15p7584s1.pdf]

Supplemental materials

Figure S1

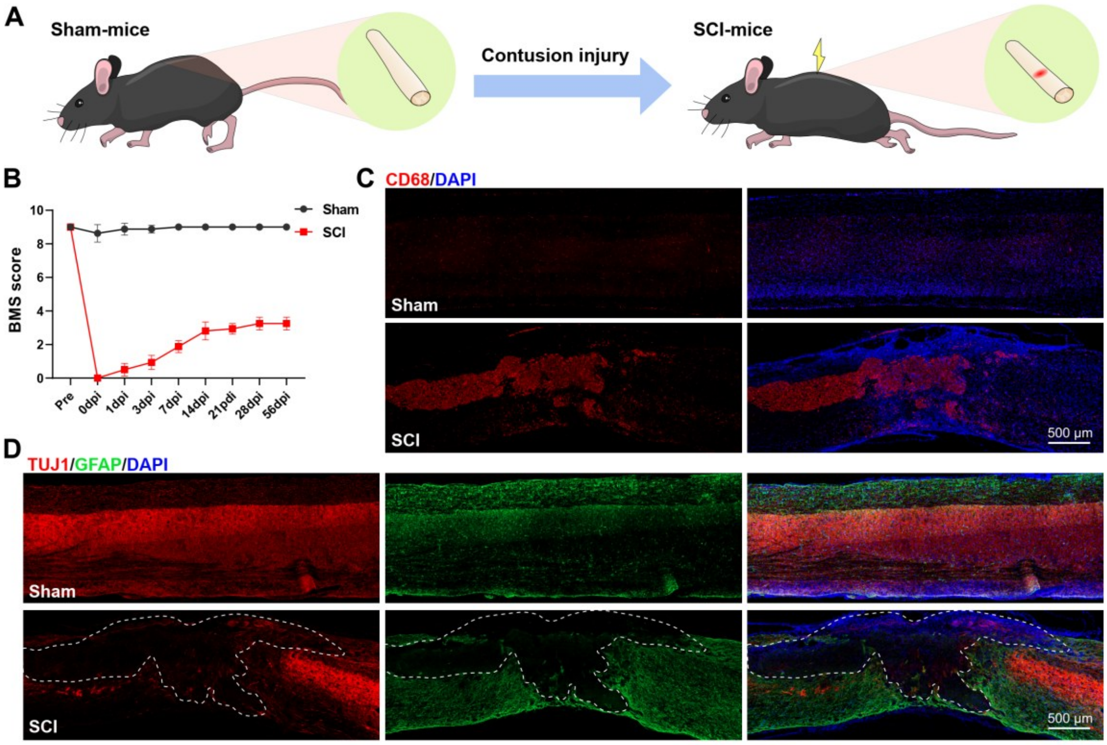

Establishion of SCI model. (A) Schematic diagram of the SCI model. (B) BMS scores in sham and SCI mice at different time points post-SCI. n = 8 per group. (C) Representative immunofluorescent stains of CD68 images of the spinal cord at 56 days post-injury in each group. Scale bar, 500 μm. (D) Representative immunofluorescent stains of TUJ1 and GFAP images of the spinal cord at 56 days post-injury in each group. Scale bar, 500 μm.

Figure S2

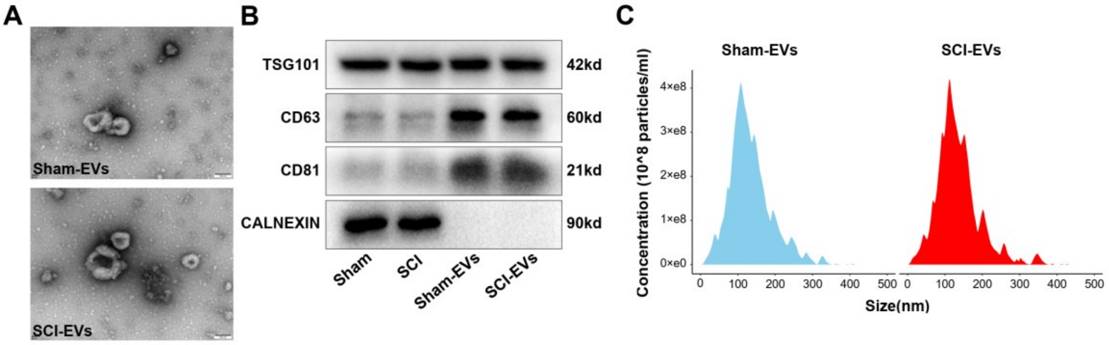

Characteristics of the EVs-derived from the injured spinal cord. (A) TEM image of EVs secreted by the sham and injured spinal cord. scale bar, 100 nm. (B) Western blotting analysis of EV-specific markers CD81, CD63, and TSG101, as well as the negative EV marker CALNEXIN. (C) Nanoparticle tracking analysis of EVs isolated from the sham and injured spinal cord.

Figure S3

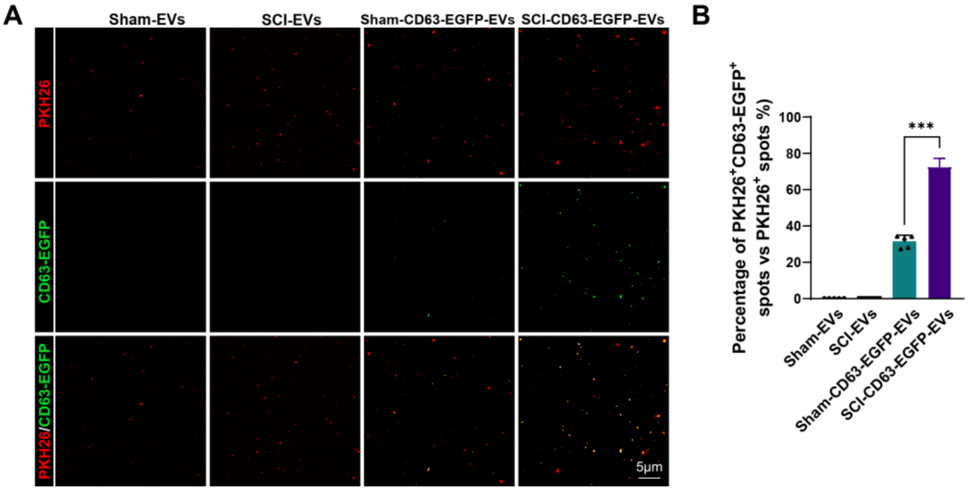

3D-τ-STED/STED-FLIM for EVs detecting. (A) Representative immunofluorescent images showed the co-labeling of PKH26 and CD63-EGFP in EVs isolated from the spinal cord in sham and SCI mice after AAV-CD63-EGFP treatment. Scale bar, 5 μm. (B) Quantification of the percentage of PKH26$^+$CD63-EGFP$^+$ spots/PKH26$^+$ spots (one-way ANOVA, F (3, 16) = 721.3, $p < 0.001$. Tukey's post hoc test. $n = 5$ per group).

Figure S4

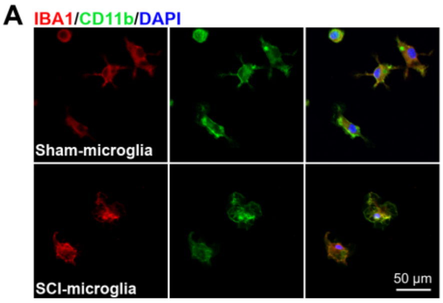

Charicteristic of sorted microglia. (A) Representative immunofluorescent images of the microglia isolated from the spinal cord of sham or SCI imce. Scale bar, 50 μm.

Figure S5

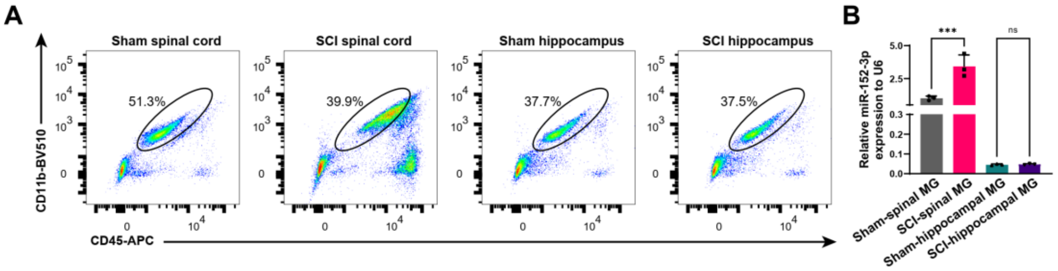

Cell sorting for spinal and hippocampal microglia. (A) Flow cytometry strategy for spinal and hippocampal microglia isolation. The DAPI^{-}/CD45^{low}/CD11b^{+} cells were sorted as microglia. (B) Quantitative analysis of the miR-152-3p expression between Sham-EVs and SCI-EVs using qRT-PCR (one-way ANOVA, F (3, 8) = 39.11, p < 0.001. Tukey's post hoc test. n = 3 per group). Data are presented as mean ± SD, NS, no significant difference, *P < 0.05, **P < 0.01, ***P < 0.001.

Figure S6

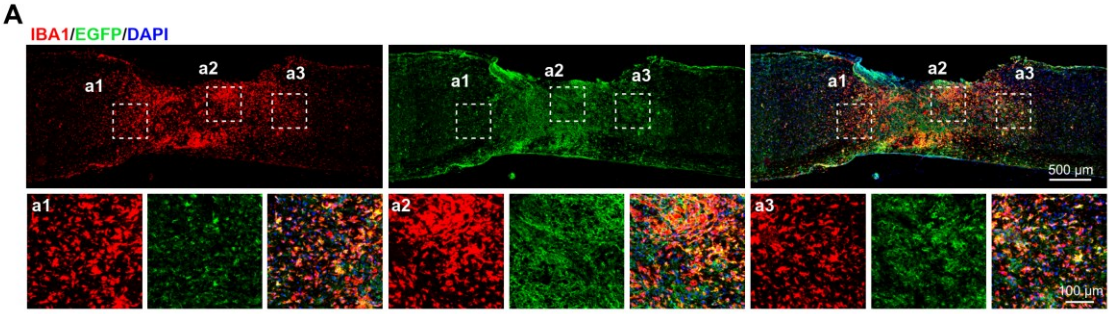

AAV-miR152-IN transfection in Cx3cr1-CreERT2 mice. (A) Immunofluorescence identification of viral transfection efficiency. Scale bar, 500 μm. (a1-a3) Enlarged images of the white box area in (A). Scale bar, 100 μm.

Figure S7

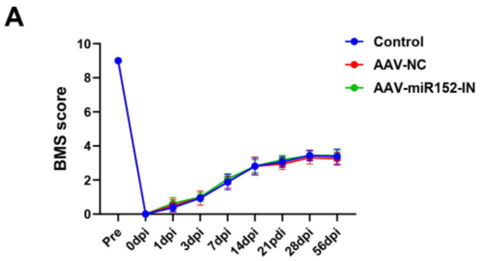

BMS scores. (A) Quantification of BMS score in control, AAV-NC and AAV-152-IN treated mice (two-way ANOVA, F (16, 168) = 0.3362, p = 0.9926. Tukey's post hoc test. n = 8 per group).

Figure S8

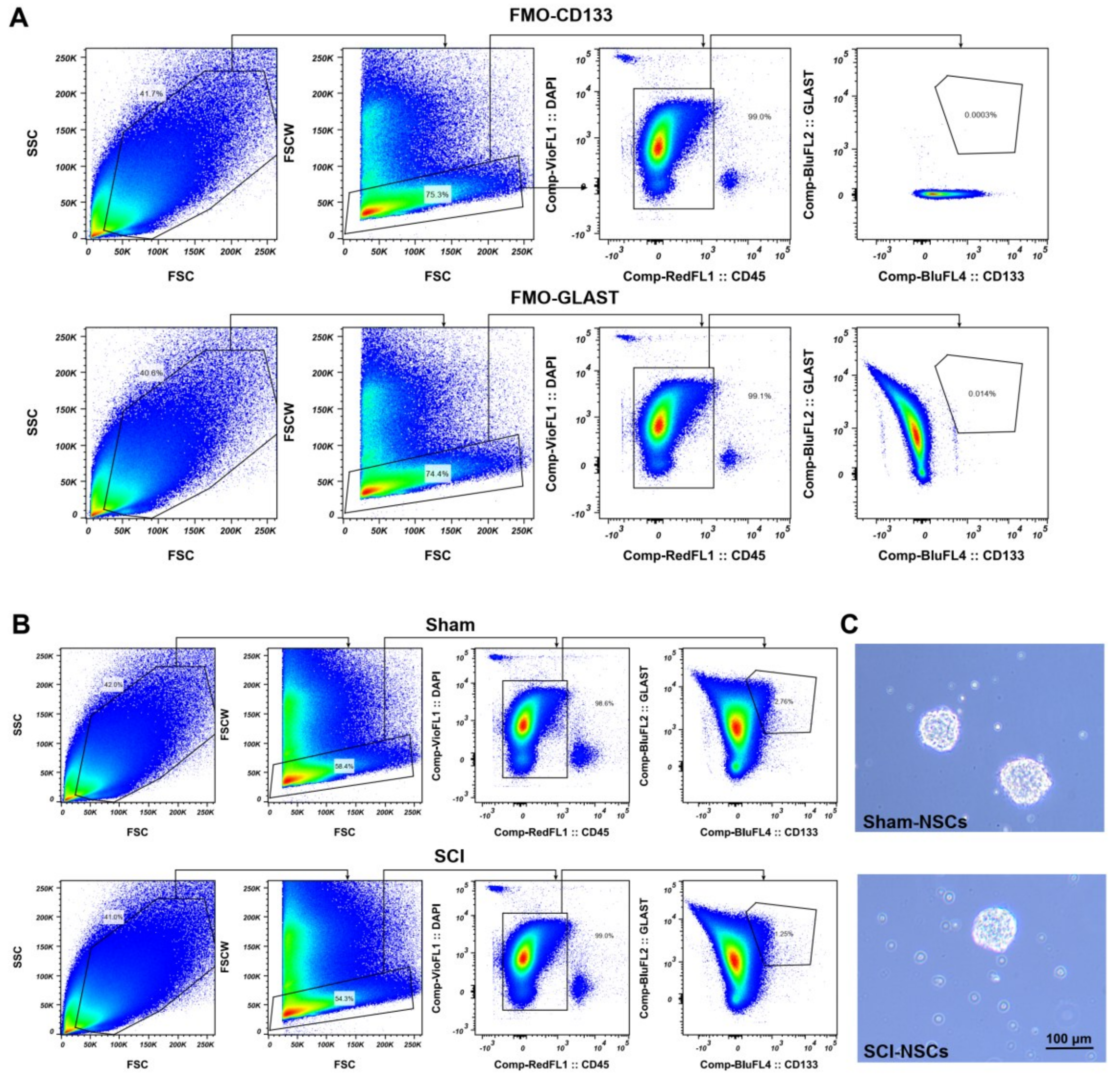

Flow cytometry cell sorting strategy for hippocampal NSCs. (A) Fluorescence Minus One (FMO) for NSCs sorting strategy to reduce the mutual interference between flow dyes. (B) Flow cytometry strategy for hippocampal NSCs isolation from sham or SCI mice. The DAPI$^-$/CD45$^-$/GLAST$^+$/CD133$^+$ cells were sorted as hippocampal NSCs. (C) Microscopic images of sorted NSCs after culturing for 5 days. Scale bar, 100 μm.

Figure S9

A

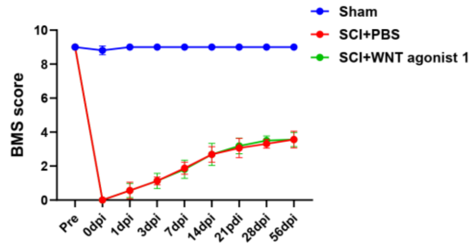

BMS scores. (A) Quantification of BMS score in sham, SCI+PBS and SCI+WNT agonist 1 groups (two-way ANOVA, F (16, 168) = 258.0, $p < 0.0001$. Tukey's post hoc test. $n = 8$ per group).

Table S1

The antibodies used in the study

**Antibody**	**Species**	**Dilution**	**Supplier**	**Catalog number**
WNT10b	Rabbit	IF: 1:200<br>WB: 1:1000	Invitrogen	PA5-116125
CD63	Mouse	IF: 1:100<br>WB: 1:1000	Abcam	ab217345
CD81	Rabbit	WB: 1:1000	Zenbio	R381296
TSG101	Rabbit	WB: 1:1000	Proteintech	28283-1-AP
CALNEXIN	Rabbit	WB: 1:1000	Proteintech	10427-2-AP
β-CATENIN	Rabbit	WB: 1:1000	Abcam	ab32572
LAMP2	Rabbit	WB: 1:1000	Abcam	ab13524
RAB27	Mouse	WB: 1:1000	Proteintech	66058-1-Ig
CD9	Mouse	WB: 1:1000	Abcam	ab2215
TUJ1	Rabbit	IF: 1:400	Abcam	ab78078
SOX2	Mouse	IF: 1:500	Abcam	ab79351
BRDU	Rabbit	IF: 1:500	Abcam	ab152095
DCX	Rabbit	IF: 1:500	Cell Signaling Technology	56130
IBA1	Rabbit	IF: 1:500	FUJIFILM Wako	019-197414987481428584
GFAP	Goat	IF: 1:500	Abcam	ab53554
CD11b-Percp-cy5.5	Rat	Flow: 1:200	Biolegend	101228
CD11b-BV510	Rat	Flow: 1:200	Biolegend	101245
CD45-APC	Rat	Flow: 1:200	Biolegend	103112
CD133-Percp-eFlour710	Rat	Flow: 1:100	Invitrogen	46-1331-82
GLAST-PE	Rat	Flow: 1:50	Miltenyi	130-118-344
ACTIN	Rabbit	WB: 1:20000	Huabio	ET1607-53
Anti-Rabbit (HRP)	Goat	WB: 1:5000	Abcam	ab6721
Anti-Rabbit 647	Donkey	IF: 1:1000	Abcam	ab150079
Anti-Rabbit 488	Donkey	IF: 1:1000	Abcam	ab150073
Anti-Rabbit 594	Donkey	IF: 1:1000	Abcam	ab150076
Anti-Goat 488	Donkey	IF: 1:1000	Abcam	ab150129
Anti-Mouse 488	Donkey	IF: 1:1000	Abcam	ab150105

WB: Western blot, IF: Immunofluorescence, Flow: Flow cytometry

Table S2

Primers for qRT-PCR

**Primers**	**Sequence (5'–3')**
Cd63-Forward	GAAGCAGGCCATTACCCATGA
Cd63-Reverse	TGACTTCACCTGGTCTCTAAACA
Lamp2-Forward	TGTATTTGGCTAATGGCTCAGC
Lamp2-Reverse	TATGGGCACAAGGAAGTTGTC
Rab27-Forward	TCGGATGGAGATTACGATTACCT
Rab27-Reverse	TTTTCCCTGAAATCAATGCCCA
Cd9-Forward	ATGCCGGTCAAAGGAGGTAG
Cd9-Reverse	GCCATAGTCCAATAGCAAGCA
miR-199a-5p	ATCCCAGTGTTCAGACTACCTGTTC
miR-483-5p	AAGACGGGAGAAGAGAAGGGAG
miR-199a-3p	GGCACAGTAGTCTGCACATTGGTTA
miR-351-5p	ATATCCCTGAGGAGCCCTTTGAG
miR-503-5p	ATTATAGCAGCGGGAACAGTACTGCAG
miR-223-3p	CGCTGTCAGTTTGTCAAATACCCCA
miR-199b-3p	CCGACAGTAGTCTGCACATTGGTTA
miR-322-3p	AAACATGAAGCGCTGCAACAC
miR-152-3p	TGCTCAGTGCATGACAGAACTTGG
miR-199b-5p	TGCCCCAGTGTTTAGACTACCTGTTC
Pri-152-3p	Ribobio China
U6 Primer	Ribobio China
Universal miRNA Primer Reverse	Ribobio China
GAPDH-Forward	AATGGATTTGGACGCATTGGT
GAPDH-Reverse	TTTGCACTGGTACGTGTTGAT
